# A two‐tier bioinformatic pipeline to develop probes for target capture of nuclear loci with applications in Melastomataceae

**DOI:** 10.1002/aps3.11345

**Published:** 2020-05-09

**Authors:** Johanna R. Jantzen, Prabha Amarasinghe, Ryan A. Folk, Marcelo Reginato, Fabian A. Michelangeli, Douglas E. Soltis, Nico Cellinese, Pamela S. Soltis

**Affiliations:** ^1^ Department of Biology University of Florida Gainesville Florida 32611 USA; ^2^ Florida Museum of Natural History University of Florida Gainesville Florida 32611 USA; ^3^ Department of Biological Sciences Mississippi State University Starkville Mississippi 39762 USA; ^4^ Institute of Systematic Botany The New York Botanical Garden Bronx New York 10458 USA; ^5^ Universidade Federal do Rio Grande do Sul Porto Alegre Rio Grande do Sul 90040‐060 Brazil

**Keywords:** HybPiper, MarkerMiner, *Memecylon*, phylogenomics, target capture, *Tibouchina*

## Abstract

**Premise:**

Putatively single‐copy nuclear (SCN) loci, which are identified using genomic resources of closely related species, are ideal for phylogenomic inference. However, suitable genomic resources are not available for many clades, including Melastomataceae. We introduce a versatile approach to identify SCN loci for clades with few genomic resources and use it to develop probes for target enrichment in the distantly related *Memecylon* and *Tibouchina* (Melastomataceae).

**Methods:**

We present a two‐tiered pipeline. First, we identified putatively SCN loci using MarkerMiner and transcriptomes from distantly related species in Melastomataceae. Published loci and genes of functional significance were then added (384 total loci). Second, using HybPiper, we retrieved 689 homologous template sequences for these loci using genome‐skimming data from within the focal clades.

**Results:**

We sequenced 193 loci common to *Memecylon* and *Tibouchina*. Probes designed from 56 template sequences successfully targeted sequences in both clades. Probes designed from genome‐skimming data within a focal clade were more successful than probes designed from other sources.

**Discussion:**

Our pipeline successfully identified and targeted SCN loci in *Memecylon* and *Tibouchina*, enabling phylogenomic studies in both clades and potentially across Melastomataceae. This pipeline could be easily applied to other clades with few genomic resources.

Inferring robust molecular phylogenies for large clades is important for understanding evolutionary patterns across the tree of life. However, harvesting suitable molecular data sets to generate these phylogenies can be challenging. Current methods for identifying single‐copy nuclear (SCN) loci rely on transcriptomic and genomic resources. Melastomataceae exemplify large clades that lack well‐curated genomic resources. Hence, SCN markers have been underutilized in phylogenetic analyses of such groups. We identify and evaluate SCN loci for two distantly related lineages of Melastomataceae to address evolutionary questions at inter‐ and intraspecific levels.

Melastomataceae (Myrtales), comprising Melastomatoideae (~5020 species) and Olisbeoideae (450–480 species), are among the 10 largest angiosperm families (Angiosperm Phylogeny Group, 2016; Christenhusz and Byng, 2016). Although the monophyly of Melastomataceae is well supported, a global phylogenetic hypothesis is lacking and the backbone phylogeny remains unresolved or poorly supported (Renner, 1993, 2004; Clausing and Renner, 2001; Renner and Meyer, 2001; Stone, 2006; Berger et al., 2016). Within Melastomataceae, the backbones of clade‐ or genus‐specific phylogenies also remain unresolved (Michelangeli et al., 2004, 2008; Stone, 2006; Goldenberg et al., 2008; Penneys et al., 2010; Bacci et al., 2019); multiple paraphyletic groups have been detected, although well‐supported, diagnosable clades have also been recovered (Goldenberg et al., 2008; Penneys and Judd, 2011; Michelangeli et al., 2013; Reginato and Michelangeli, 2016a; Rocha et al., 2016; Bacci et al., 2019).

Resolving the backbone phylogeny of Melastomataceae remains challenging because of the continued use of only a few plastid and nuclear loci, often reusing the same sequences while adding new accessions (Clausing and Renner, 2001; Renner and Meyer, 2001). Researchers are starting to develop SCN markers, although their use across Melastomataceae has been limited (Reginato and Michelangeli, 2016b; Dai et al., 2019). In addition, obtaining the quantity and quality of DNA needed for sequencing is hampered by the presence of secondary metabolites, often requiring repeated extractions and/or sequencing (Renner et al., 2001).

With the development of next‐generation sequencing and phylogenomic tools (e.g., Chamala et al., 2015; Johnson et al., 2016), many of these challenges can be successfully addressed. These methods are well‐equipped to deal with low‐quantity and poor‐quality DNA. However, the limited number of transcriptomes and lack of a sequenced genome within Melastomataceae have hindered the application of phylogenomic approaches. To date, two transcriptomes have been sequenced from Melastomataceae (*Miconia* *bicolor* (Mill.) Triana [syn. *Tetrazygia bicolor* (Mill.) Cogn.] and *Medinilla magnifica* Lindl.) through the 1KP initiative (http://www.onekp.com). These available resources are from Melastomatoideae, and their utility for applications more broadly within Melastomatoideae or in Olisbeoideae is unclear. A probe set of 353 nuclear genes for targeted sequencing across angiosperms is available, but *M. bicolor* and *M. magnifica* were the only representatives from Melastomataceae used to design and test these probes (Johnson et al., 2019). The sequence similarity of probe sequences designed from these two transcriptomes and target sequences in distantly related clades within Melastomataceae is likely low. Therefore, success in targeting loci in distantly related taxa within the family using standard probe design approaches is uncertain. To increase the likelihood of successfully targeting loci in distantly related clades of Melastomataceae, we optimized probe design using low‐cost genome‐skimming data from taxa closely related to two clades of interest within the family. Using closely related genome‐skimming reads to ensure high sequence similarity between probes and target sequences should increase the enrichment and sequencing success of loci for phylogenomic analysis.

We designed a two‐tiered pipeline to identify and target SCN loci for taxa with few genomic or transcriptomic resources for phylogenetic inference in two clades of interest in both Melastomatoideae (*Tibouchina* Aubl.) and Olisbeoideae (*Memecylon* L.). First, two publicly available transcriptomes from Melastomataceae outside *Tibouchina* and *Memecylon* were used to identify putatively SCN loci based on two annotated angiosperm genomes using MarkerMiner (Chamala et al., 2015). In the second tier, we assembled suitable template sequences for probe design for these loci from within the clades of interest using genome‐skimming reads. This study will facilitate research in Melastomataceae by identifying loci for generating a robust phylogenetic framework necessary for macroevolutionary, biogeographic, and systematic studies. Our work may serve as a model for the application of this pipeline in other groups with few genomic resources.

## METHODS

**Figure 1:**
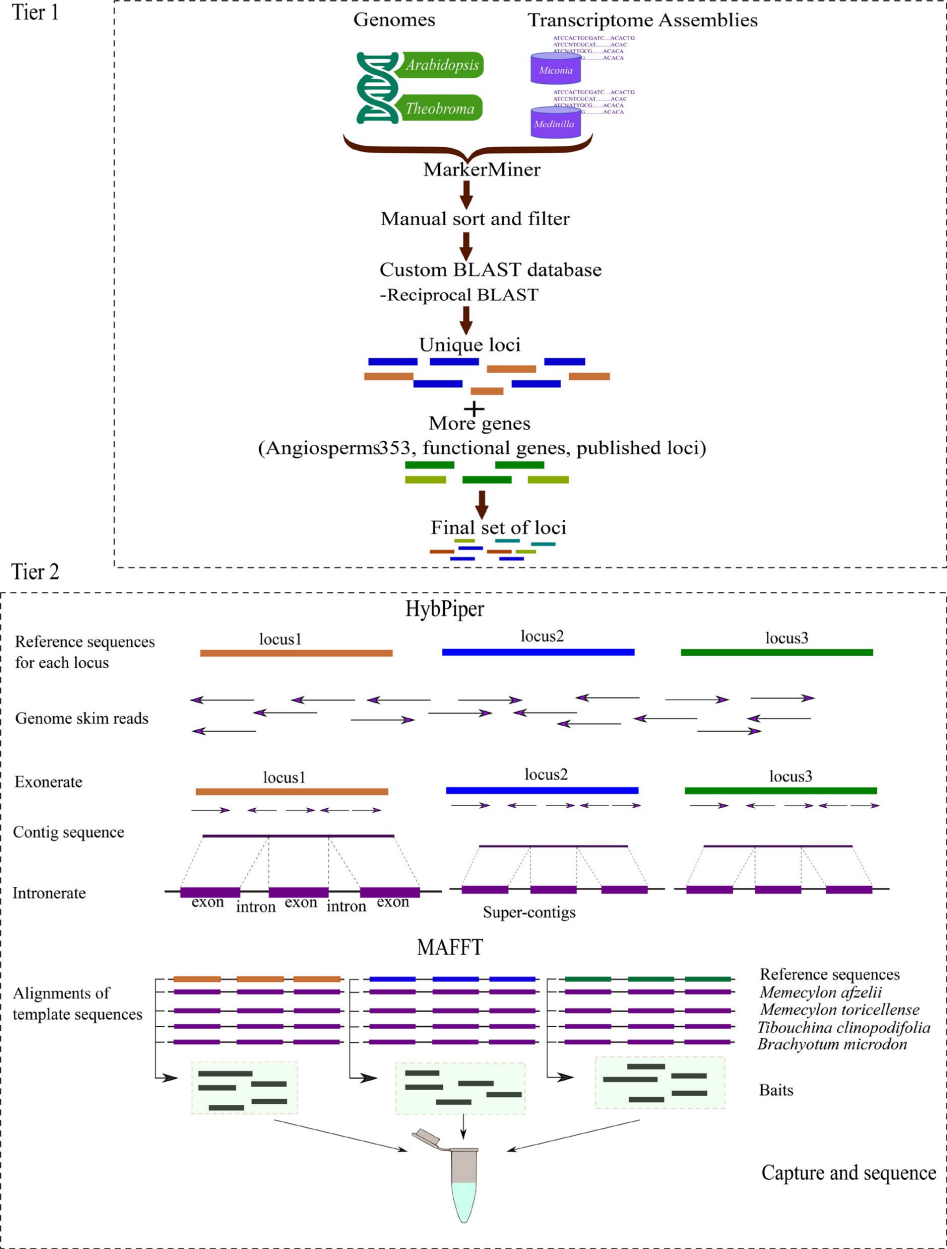
Overview of the steps in the two‐tiered probe development pipeline. In the first tier, loci are selected using MarkerMiner and loci from other sources are added. In the second tier, genome‐skimming reads are assembled using HybPiper to the reference sequence for each locus selected using the first tier; alignments of assembled sequences were used for probe design for target capture.

The detailed workflows of our two‐tiered approach (Fig. 1) are described below.

### Genome skim sequencing

To facilitate probe design, genome‐skimming data sets were constructed for two species each of Olisbeoideae (*Memecylon afzelii* G. Don and *M. torricellense* Lauterb.) and Melastomatoideae (both from Melastomateae: *Tibouchina clinopodifolia* (DC.) Cogn. and *Brachyotum microdon* (Naudin) Triana). For the Olisbeoideae samples, total genomic DNA was extracted from herbarium samples (*M. afzelii*: van der Burgt et al. 947 [MO], *M. torricellense*: Takeuchi and Ama 16241 [MO]) following Doyle and Doyle (1987). DNA was quantified using a Qubit DNA BR assay (Thermo Fisher Scientific, Waltham, Massachusetts, USA). Total genomic library preparation and barcoding were performed by RAPiD Genomics (Gainesville, Florida, USA) for sequencing on a HiSeq 3000 platform (Illumina, San Diego, California, USA) with 150‐bp paired‐end reads, with a sequencing effort corresponding to 15× coverage of a nuclear genome the size of *Eucalyptus grandis* W. Hill (Myrtaceae), the closest reference genome. Raw FASTQ reads were trimmed and adapters removed using Trimmomatic 0.33 (Bolger et al., 2014) with a sliding window of 20 bp and quality score of Q20 or greater, and the final quality of reads was assessed using FastQC (Andrews, 2010).

For Melastomateae samples, total genomic DNA was isolated from silica‐dried tissue using the QIAGEN DNAeasy Plant Mini Kit (QIAGEN, Valencia, California, USA) following Alexander et al. (2007). Total DNA samples were quantified using a NanoDrop Spectrophotometer (Thermo Fisher Scientific). Total genomic libraries and barcoding were performed at Cold Spring Harbor Laboratories for sequencing on a HiSeq 2000 with 100‐bp paired‐end reads. Putative non‐ribosomal nuclear read pairs were recovered (11,171,050 for *B. microdon* and 21,273,218 for *T. clinopodifolia*) from a genome‐skimming sequencing run after subtracting putative plastid and mitochondrial reads (Illumina). Paired‐end reads were then imported into Geneious 7.1 (Biomatters Ltd., Auckland, New Zealand) and trimmed by quality score using the default parameters for the modified Mott algorithm at 0.05 probability.

### Tier 1

Putatively SCN loci were identified with MarkerMiner 1.0, which uses reciprocal BLAST searches of input transcriptome sequences and curated lists of putatively single‐copy loci from reference genomes (De Smet et al., 2013; Chamala et al., 2015). The reference genomes used were *Arabidopsis thaliana* (L.) Heynh. (Gan et al., 2011) and *Theobroma cacao* L. (Argout et al., 2011), and the transcriptomes used were *Miconia bicolor* (sample ID: SWGX) and *Medinilla magnifica* (sample ID: WWQZ) from 1KP (Matasci et al., 2014). With a minimum gene length of 500 bp enforced, we identified 948 putatively SCN loci based on the *A. thaliana* genome and 1045 loci based on the *T. cacao* genome.

**Figure 2:**
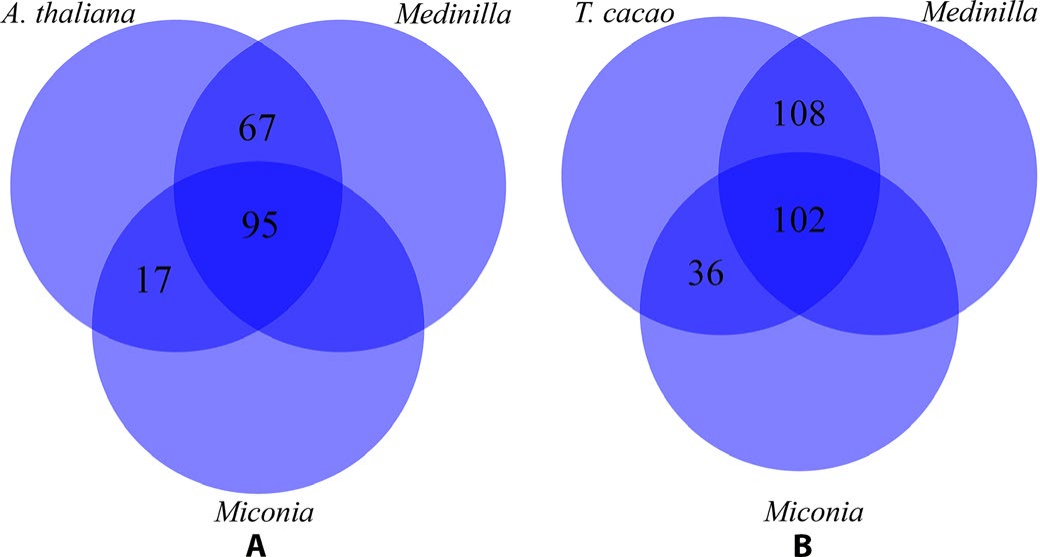
Venn diagrams of transcriptomic (*Miconia* and *Medinilla*) sources of loci identified by MarkerMiner (from Tier 1) by a genomic resource. (A) Loci common to the *Arabidopsis thaliana* genome and the *Medinilla* and *Miconia* transcriptomes. (B) Loci common to the *Theobroma cacao* genome and the *Medinilla* and *Miconia* transcriptomes. The comparison of overlap between genomes is not visualized.

Loci were manually sorted, trimmed, and filtered to retain loci containing individual exon sequences longer than 120 bp and individual intron sequences shorter than 100 bp to ensure sequence capture across introns using 120‐bp probes with 3× tiling. After filtering, 95 loci from *A. thaliana* and 102 loci from *T. cacao* were identified in both transcriptomes, including 61 loci common to both genomes; the longer transcriptome sequence was selected as the representative for the locus to avoid ambiguous nucleotides for probe design, with the *Miconia bicolor* transcriptome sequence chosen by default if sequences were of the same length. Loci found in only one reference transcriptome were also retained (see Fig. 2). This filtering identified 425 suitable loci, including overlapping loci, each represented by one Melastomataceae transcript. Custom databases were constructed from these transcripts in Geneious version 10.2 (Kearse et al., 2012) to conduct reciprocal BLAST searches to identify overlapping loci identified from both genomes (Altschul et al., 1990). This reciprocal BLAST search was conducted several times: (1) for those loci found in both transcriptomes, (2) for those loci found in only a single transcriptome, and (3) for all loci together. Where loci were identified as overlapping due to retrieval from both genomes, the longer transcript was retained as the representative sequence for the locus; for sequences of the same length, the default was the transcript identified by the *T. cacao* genome. Although a reciprocal BLAST step may identify potential paralogs, only loci identified from both genomes (and no potential paralogs) were detected and reduced to a single representative. Following this reciprocal BLAST, 30 loci were identified as unique to the *A. thaliana* data set, 39 loci were unique to the *T. cacao* data set, and 62 were recovered from both genomes, resulting in a total of 131 loci found in both transcriptomes. Including loci found in only one of the transcriptomes, the final MarkerMiner data set contained 265 loci, each represented by a single transcriptome sequence. Due to the paucity of genomic resources within Melastomataceae, we were unable to confirm the single‐copy status of these loci; our single‐copy assessment relies on MarkerMiner analyses of genomic resources outside the clade.

To increase the number of loci for enrichment, loci identified as putatively single‐copy, protein‐coding, and conserved across angiosperms in the Angiosperms353 project were also included (Johnson et al., 2019). Sequences were retrieved from GitHub (https://github.com/mossmatters/Angiosperms353) and then filtered by taxon source to include only those loci that were represented by sequences from taxa in the rosids, including the two melastomes (*Miconia* and *Medinilla*), resulting in 266 loci.

To facilitate merging our data set with existing molecular matrices in Melastomataceae, we included eight putatively SCN loci developed by Reginato and Michelangeli (2016b) for use across Melastomataceae. *Miconia* sequences were retrieved from GenBank for these loci (accessions: KT377078.1, KT377070.1, KT377118.1, KT377126.1, KT377110.1, KT377086.1, KT377102.1, KT377094.1). We also included a set of six multicopy loci implicated in trichome development in *A. thaliana* (Rhee, 2003; Berardini et al., 2015). A BLAST search was conducted for GenBank sequences of *A. thaliana* (accessions: AJ133743.1, NM_113708.2, NM_124699.3, NM_148067.4, NM_179236.3, NM_001198514.1) against the *Medinilla magnifica* and *Miconia bicolor* transcriptomes to obtain corresponding transcriptome sequences for these loci, one of which was selected as the representative sequence for each locus following the criteria described above. Overall, we included 265 MarkerMiner loci, 266 Angiosperms353 loci, eight published SCN loci, and six functional multicopy loci, resulting in a total of 545 loci for use in the second tier of the pipeline.

### Tier 2

We assembled homologous sequences for the loci identified in the first tier, including the Angiosperms353 loci, functional loci, and previously published loci, using genome‐skimming data from *Memecylon* and Melastomateae to generate clade‐specific template sequences for probe design. We make the distinction between loci (regions of the genome or “genes”) and template sequences (one of several versions of the nucleotide sequence for each locus).

Sequences from the first tier were used as reference sequences for assembly in HybPiper. Genome‐skimming reads for *Memecylon afzelii*,* M. torricellense*,* Tibouchina clinopodifolia*, and *Brachyotum microdon* were assembled against these reference sequences. Read assembly was conducted for both paired and unpaired reads in HybPiper version 1.3.1 with the Burrows–Wheeler alignment method of aligning reads to targets (Li and Durbin, 2009; Johnson et al., 2016). For the MarkerMiner loci, flanking intronic regions adjacent to the targeted exons were also assembled. For each locus, we then retrieved and aligned the assembled sequences with the reference sequence using MAFFT version 7.215 and the default parameters (Katoh and Standley, 2013).

For each locus, we selected between one and four sequences from the alignment based on relative sequence length and sequence similarity for use as template sequences for probe design. For loci with less than 90% sequence identity between the transcriptome and genome‐skimming sequences or between individual genome‐skimming sequences, multiple sequences were retained as templates. Genome‐skimming contigs were typically shorter than transcriptome contigs. To ensure full‐length coverage of loci, genome‐skimming sequences were occasionally joined with the homologous transcriptome or genome‐skimming sequences to form a hybrid sequence; this maximized capture success because divergent regions reduce the likelihood of capture. MarkerMiner loci that were not assembled from any genome‐skimming data sets were omitted. For several loci, the recovered contigs were less than 300‐bp long; therefore, the transcriptome sequence was included as the sole representative for probe design, even though a contig had been recovered from the genome‐skimming data sets. In the final data set, each locus was represented by between one and four template sequences from various sources (see Table 1), and therefore, between one and four sets of probes were designed for each locus to ensure successful enrichment for both *Memecylon* and *Tibouchina*.

**Table 1:** Sources of loci and template sequences by method and genome source.

Locus sources (identification method)	Genomic sources for template sequences
MarkerMiner	*Miconia* transcriptome
Angiosperms353	*Medinilla* transcriptome
Published SCN (Reginato and Michelangeli, 2016b)	*Memecylon* genome skims (two species)
Functional loci (TAIR database)	Melastomateae genome skims (two species)
	*Miconia* GenBank sequences
	Angiosperms353 sequences (rosids)

To ensure that no plastid or mitochondrial loci were targeted, a BLAST search of these final template sequences was conducted against the plastid genome of *Tibouchina longifolia* (Vahl) Baill. (accession: KX826833.1) and against the most closely related sequenced mitochondrial genome available (*Lagerstroemia indica* L. [Myrtales]; accession: NC_035616.1). We selected 384 loci represented by 689 template sequences for probe design (155 loci with one template; 164 loci with two templates; 54 loci with three templates; 11 loci with four templates). Of these, 104 loci originated from the MarkerMiner selection process, while 266 were included from the Angiosperms353 project, eight were previously published SCN loci, and six were loci of functional significance.

### Probe construction

The 689 template sequences from Tier 2, as alignments for loci with multiple template sequences, were used for probe construction. These 689 template sequences for 384 loci were used to synthesize a custom probe library of 11,871 biotinylated 120‐bp RNA probes with 3× coverage to query genomic DNA libraries for *Memecylon* and *Tibouchina* at RAPiD Genomics. Their proprietary workflow of probe construction considered the diversity of taxa so that the resulting final probe panel maximized specificity across a broad phylogenetic range. The probes were screened against the mitochondrial genome of *L. indica*, the plastid genome of *T. longifolia*, and the nuclear genome of *E. grandis* (accession: GCF_000612305.1). These probes were also screened for large homopolymers; gaps introduced through the alignment process were filtered from the template sequences. Any remaining ambiguous bases were resolved using the most frequent base in the alignment.

### Taxon sampling

The clades of interest in this study are *Memecylon* and *Tibouchina* sensu stricto (s.s.). We sequenced 93 samples of *Memecylon*, 62 of which represented major lineages and geographic areas, prioritizing samples from South Asia, Southeast Asia, and Pacific regions. We included one sample of the genus *Mouriri* Aubl. as an outgroup for *Memecylon*. Individuals belonging to the South African *Buxifolia* clade of *Memecylon* (37 samples from five taxa) were sequenced to investigate relationships within this poorly understood complex. We sequenced 144 samples of *Tibouchina*, focusing on *Tibouchina* s.s. (35 taxa), with most species represented by multiple accessions, and five outgroup taxa. Tissue samples were taken from silica‐dried field collections or herbarium specimens (*Memecylon* collections: FLAS, NY, MO, US, NSW; *Tibouchina* collections: NY, RB, HUFU, HRCB, UEC, MBM, BHCB; accession information available in Appendix S1).

### Library preparation

Total genomic DNA was extracted from herbarium samples and silica‐dried leaf tissue using a modified cetyltrimethylammonium bromide (CTAB) protocol (Doyle and Doyle, 1987; see Appendix S2 for modified protocol). To isolate enough DNA for sequencing, multiple extractions were performed for each sample, pooled, and concentrated by vacuum centrifugation. Total DNA was quantified using a Qubit BR assay. DNA quality was evaluated using the NanoDrop 2000 (Thermo Fisher Scientific) and by gel electrophoresis. Library preparation and sequence capture were performed by RAPiD Genomics utilizing their high‐throughput workflow with proprietary chemistry. Samples were multiplexed, and the captured fragments were pooled and sequenced on an Illumina HiSeq 3000 platform with 150‐bp paired‐end reads.

### Sequence cleaning and assembly

Raw reads were quality‐filtered and trimmed to remove adapter sequences and low‐quality reads with Trimmomatic (Bolger et al., 2014) using scripts from the Sequence Capture Processor pipeline with the default parameters, with the following modifications: a simple adapter clip threshold with a minimum score of 5, a minimum score of 20 for the palindrome clip threshold, a maximum of 5 mismatches allowed in the seed, and cleaving 10 bases from the start of the reads (Andermann et al., 2018). Cleaned reads were assembled with HybPiper version 1.3.1 using the template sequences used for probe design as references (Johnson et al., 2016). Assembly was conducted for all reference sequences together, where each locus was represented by up to four different template sequences. Summary statistics were obtained using HybPiper scripts, except for intron and supercontig sequence lengths, and percent sequence identity between template and captured sequences, which were calculated using custom scripts. Templates are considered to have recovered sequences if indicated by the HybPiper exonerate.py script using default similarity and length thresholds (Johnson et al., 2016). Potential paralogs, as indicated by the presence of multiple long contigs for a locus, were identified by HybPiper scripts. Percent identity as calculated here includes gaps and trailing ends of sequences. Statistics were summarized by species, template sequence, locus, and clade using custom R scripts.

To compare sequencing and assembly success for the Angiosperms353 loci with published data, and to identify whether recovery failure was at the biochemical or bioinformatic level, assembly for the Angiosperms353 loci was also conducted using homologous amino acid sequences from the 1KP capstone paper using the BLASTX algorithm in HybPiper (Johnson et al., 2019; One Thousand Plant Transcriptomes Initiative, 2019). Using amino acid sequences as references for assembly can allow for greater sequence divergence and therefore may improve assembly success for highly divergent taxa. All custom scripts, template sequences, probes, and links to publicly available code are on GitHub (https://github.com/jjantzen/Probe_design) and Dryad (https://doi.org/10.5061/dryad.8931zcrm2; Jantzen et al., 2020).

## RESULTS

We present the percent on‐target reads, read counts, read depth, number of loci for which sequences were recovered, and number of potential paralogs for representative species (Table 2), for the population‐level sampling of *Memecylon* (Table 3), and averaged for each clade (Table 4; full results presented in Appendix S3). For each locus, mean, minimum, and maximum total lengths of exons, introns, and supercontigs, the number of taxa successfully sequenced, and the percent identity between recovered sequences and each template sequence are presented in Appendix S4, and are summarized by the genomic source of the template in Table 5.

**Table 2:** Sequencing statistics for target enrichment of representative species of *Memecylon* and *Tibouchina*, averaged when multiple samples were sequenced per species.

Species	Percent on‐target reads	No. of total reads	Mean locus length per species (excluding zeros)	Maximum locus length per species	No. of templates with sequences (min–max)	No. of templates with sequences at 50% of reference length (min–max)	No. of loci with potential paralogs
*Memecylon australissimum*	84.6	4,060,390	551	4314	332 (329–334)	218 (207–229)	4
*Memecylon bachmannii*	86.6	4,306,749	539	4698	323 (290–347)	209 (180–229)	4
*Memecylon flavescens*	79.8	2,625,799	536	4230	306	198	8
*Memecylon hookeri*	83.9	1,271,186	553	4227	288	192	2
*Memecylon macrophyllum*	80.8	1,540,585	552	4224	295	193	2
*Memecylon maxwellii*	92.9	2,146,671	493	4233	305	186	4
*Memecylon natalense*	83.1	2,127,129	555	4314	310 (289–334)	207 (177–226)	2
*Memecylon rhinophyllum*	84.2	2,281,370	559	4230	305	205	5
*Memecylon rivulare*	89.6	2,600,174	530	4338	311	196	1
*Memecylon soutpansbergense*	76.1	821,062	572	4314	290	202	3
*Memecylon* sp7	73.3	2,657,756	529	4104	318	203	15
*Memecylon umbellatum*	85.9	2,332,960	545	4092	315 (301–328)	206 (201–211)	5
*Mouriri helleri*	88.7	1,536,920	516	4215	311	187	3
*Tibouchina aegopogon*	81.4	1,774,516	550	4302	349 (295–366)	261 (219–275)	44
*Tibouchina aspera*	87.4	3,226,663	566	3969	348 (243–402)	262 (154–323)	14
*Tibouchina barbigera*	86.1	2,344,437	571	4512	364 (312–394)	279 (222–316)	51
*Tibouchina catharinae*	89.8	1,273,246	527	3840	324 (312–335)	235 (228–241)	8
*Tibouchina gracilis*	68.0	2,389,578	591	3831	320	262	21
*Tibouchina karstenii*	89.7	1,560,620	523	3831	346 (339–353)	243 (239–247)	28
*Tibouchina llanorum*	87.6	2,048,631	532	3840	328 (265–382)	241 (197–280)	35
*Tibouchina papyrus*	83.2	3,072,283	549	3891	364 (338–397)	271 (232–321)	61
*Tibouchina striphnocalyx*	84.7	5,222,221	574	3837	360	277	25

**Table 3:** Sequencing statistics for target enrichment of population‐level sampling of *Memecylon*.

Sample	Species	No. of total reads	Percent on‐target reads	No. of templates with sequences	No. of templates with sequences at 50% of reference length	No. of potential paralogs
C3	*M. bachmannii*	2,956,804	84.0	316	215	2
E3	*M. bachmannii*	5,766,861	86.5	328	199	3
E5	*M. bachmannii*	973,415	86.6	296	189	1
G3	*M. bachmannii*	2,845,352	90.1	320	205	3
GM5	*M. bachmannii*	2,166,529	89.4	306	197	3
MK3	*M. bachmannii*	12,565,563	87.6	350	225	8
MK6	*M. bachmannii*	798,693	88.9	290	180	0
OM10	*M. bachmannii*	5,766,164	83.8	334	222	6
OM12	*M. bachmannii*	2,796,409	87.8	320	202	5
SIL1	*M. bachmannii*	5,976,421	87.4	346	217	8
U5	*M. bachmannii*	4,217,194	86.6	329	218	3
B2	*M. natalense*	1,086,552	80.4	302	199	3
B4	*M. natalense*	2,564,946	81.8	321	216	4
L1	*M. natalense*	1,205,197	80.1	299	206	3
L3	*M. natalense*	1,917,426	87.8	312	194	5
LP2	*M. bachmannii*	4,587,459	88.8	326	210	4
M4	*M. bachmannii*	4,929,180	78.7	332	229	3
MG1	*M. bachmannii*	3,948,452	86.2	319	209	2
BR1	*M. natalense*	524,245	86.0	252	174	1
ME1	*M. natalense*	5,329,524	89.0	327	211	1
MO2	*M. natalense*	2,126,241	87.3	310	202	3
MO3	*M. natalense*	4,695,432	87.3	332	221	4
NK1	*M. natalense*	4,651,542	82.6	331	227	0
O1	*M. natalense*	1,265,648	76.0	303	209	1
O4	*M. natalense*	1,035,677	76.4	298	214	1
O5	*M. natalense*	1,025,963	79.6	294	205	0
O7	*M. natalense*	1,003,379	83.2	296	201	2
O8	*M. natalense*	1,040,441	77.9	297	202	4
OH1	*M. natalense*	744,716	88.0	289	177	1
OM1	*M. natalense*	891,562	83.4	301	203	1
S2	*M. natalense*	3,837,706	85.1	327	221	3
S7	*M. natalense*	1,141,947	81.4	298	211	0
W6	*M. natalense*	4,327,313	86.5	334	216	7

**Table 4:** Sequencing statistics for target enrichment of *Memecylon* and *Tibouchina*, averaged for each clade.

Statistics	*Memecylon*	*Tibouchina*
Mean percent on‐target reads (min–max)	84.5 (42–95)	84.3 (68–92)
Mean reads mapped	2,492,585	1,973,410
Mean total reads	2,913,061	2,334,623
Mean read depth	702×	554×
Mean locus count per species (min–max)	218 (101–263)	226 (206–247)
Mean template count per species (min–max)	304 (105–442)	377 (285–455)
Mean taxon count per locus (template)	30 (29)	24 (28)
Mean number of templates with sequences (min–max)	305 (105–347)	377 (79–411)
Mean number of templates with sequences at 50% of length (min–max)	200 (38–267)	259 (10–340)
Mean total exon length, bp	401	439
Mean total intron length, bp	822	568
Mean supercontig length, bp	1209	1019
Mean number of potential paralogs per species (min–max)	5 (0–15)	40 (7–102)

**Table 5:** Sequencing statistics for target enrichment of *Memecylon* and *Tibouchina*, grouped by genomic resource used to design probes.

Sample clades	Genome source for probe design				
	*Memecylon* genome skims	*Miconia* GenBank sequences	Angiosperms353 Rosid sequences	*Tibouchina* genome skims	*Medinilla* and *Miconia* transcriptomes
*Memecylon*					
No. of taxa per template	60/62	8/62	23/62	31/62	37/62
Average (min–max) percent identity between templates and target sequences	82.8 (10–100)	NA	30.2 (10–100)	49.8 (10–100)	39.0 (10–81.3)
Average (min–max) exon length	762 (60–4698)	NA	231 (51–1287)	341 (57–3837)	582 (69–1752)
Average (min–max) intron length	1545 (0–28,880)	NA	387 (0–3626)	697 (0–17,048)	595 (0–2964)
Average (min–max) supercontig length	2268 (95–19,522)	NA	617 (92–4280)	1029 (73–20,164)	1154 (74–3348)
Percent reads on target	67.8 (MM) / 10.6 (A353)	0.56	6.06	44.8 (MM) / 7.24 (A353)	60.3 (MM) / 0.38 (A353)
*Tibouchina*					
No. of taxa per template	26/40	17/40	13/40	37/40	33/40
Average (min–max) percent identity between templates and target sequences	50.8 (10–100)	25.2 (10.2–94.9)	39.7 (10–97.8)	81.0 (10–100)	38.0 (10–97.5)
Average (min–max) exon length	498 (57–3894)	206 (69–795)	407 (57–3540)	614 (54–5673)	522 (57–2043)
Average (min–max) intron length	475 (0–17,558)	399 (1–3609)	530 (0–10,108)	923 (0–19,505)	337 (0–6400)
Average (min–max) supercontig length	978 (69–22,850)	655 (88–4368)	931 (67–13,649)	1563 (63–21,330)	860 (83–7660)
Percent reads on target	28.5 (MM) / 3.2 (A353)	0.68	3.93	56.8 (MM) / 12.4 (A353)	33.6 (MM) / 0.96 (A353)

Note

A353 = Angiosperms353; MM = MarkerMiner.

### Sequencing and assembly success

Most samples were successfully sequenced for both *Memecylon* and *Tibouchina*. A few samples failed during library preparation, enrichment, and sequencing; failures were likely due to low‐quality DNA or the presence of secondary metabolites that may inhibit PCR, enrichment, and/or sequencing reactions. We present statistics for both templates and loci. Sequences from all 62 species of *Memecylon* were successfully recovered by probes from 82 template sequences, and at least 55 species were recovered by probes from 244 templates. Sequences from 84 loci were recovered for all 62 species when combining the success of probes designed from multiple template sequences for each locus, and sequences from 196 loci were recovered for at least 55 species. Template sequences assembled from *Memecylon* genome‐skimming data had on average 83% sequence identity to the *Memecylon* targeted sequences, whereas templates assembled from *Tibouchina* genome‐skimming data had on average 50% sequence identity with *Memecylon* targeted sequences. Due to the inclusion of gaps and trailing ends of sequences, percent identities were lower than expected based solely on overlapping base pairs.

Sequences from all 40 species of *Tibouchina* were successfully recovered by probes from 197 templates, and at least 35 species were recovered by probes from 302 templates. Sequences from 186 loci were recovered for all 40 species when combining the success of probes designed from multiple template sequences for each locus, and 210 sequences were recovered for at least 35 species. Template sequences designed from *Tibouchina* genome‐skimming data had on average 81% sequence identity to the *Tibouchina* targeted sequences, whereas templates designed from *Memecylon* genome‐skimming data had on average 51% sequence identity with *Tibouchina* targeted sequences.

The Angiosperms353 loci had higher sequence recovery success, producing longer assembled sequences, when using amino acid sequences for assembly in HybPiper with the BLASTX algorithm than when using the nucleotide sequences (Appendix S5). This was true regardless of the taxonomic source of the nucleotide template sequence.

### Clade comparisons

For *Memecylon*, 291 loci were sequenced, 88 of which were, on average, longer than 500 bp. For *Tibouchina*, 280 loci were sequenced, 90 of which were longer, on average, than 500 bp. We recovered 193 loci from over 50% of the species in both *Memecylon* and *Tibouchina*, 75 of which had, on average, sequences longer than 500 bp (Appendix S4). We identified probes from 56 template sequences, representing 37 loci, which each captured sequences greater than 500 bp for more than 50% of species in each clade. Six of these loci were captured by probes from more than one template.

**Figure 3:**
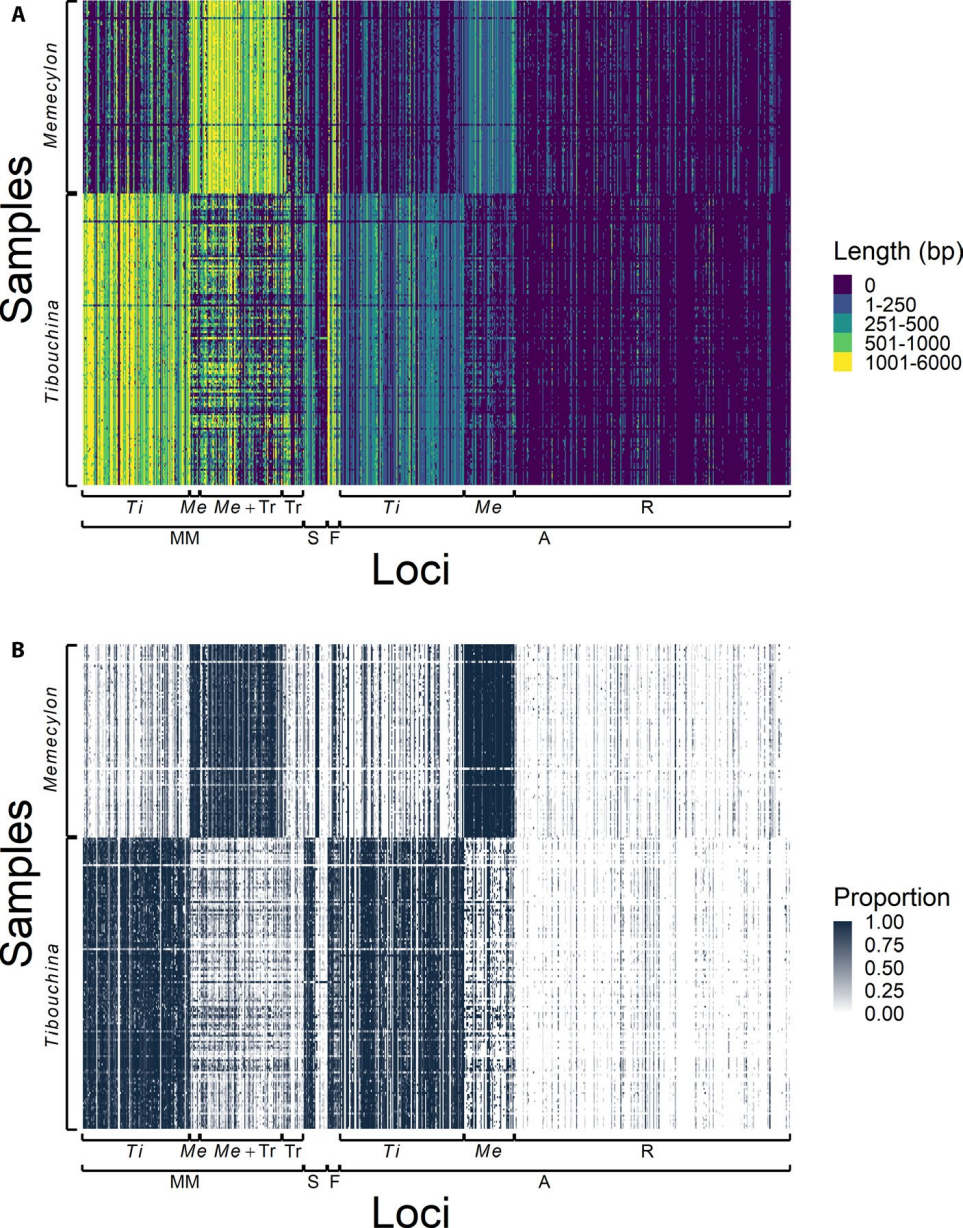
Heatmap showing sequencing success of target enrichment for *Memecylon* and *Tibouchina* (Melastomataceae). Loci are on the *x* axis, grouped first by the method of development and then by genomic source of template sequence. Taxa are on the *y* axis. (A) Colors represent length (bp) of assembled sequences. (B) Shading represents the proportion of sequence length recovered relative to the template sequence. A = Angiosperms353 loci, F = functional loci, *Me* = *Memecylon*, MM = MarkerMiner loci, R = rosids, S = published SCN loci (Reginato and Michelangeli, 2016b), *Ti* = *Tibouchina*, Tr = transcriptomes.

**Figure 4:**
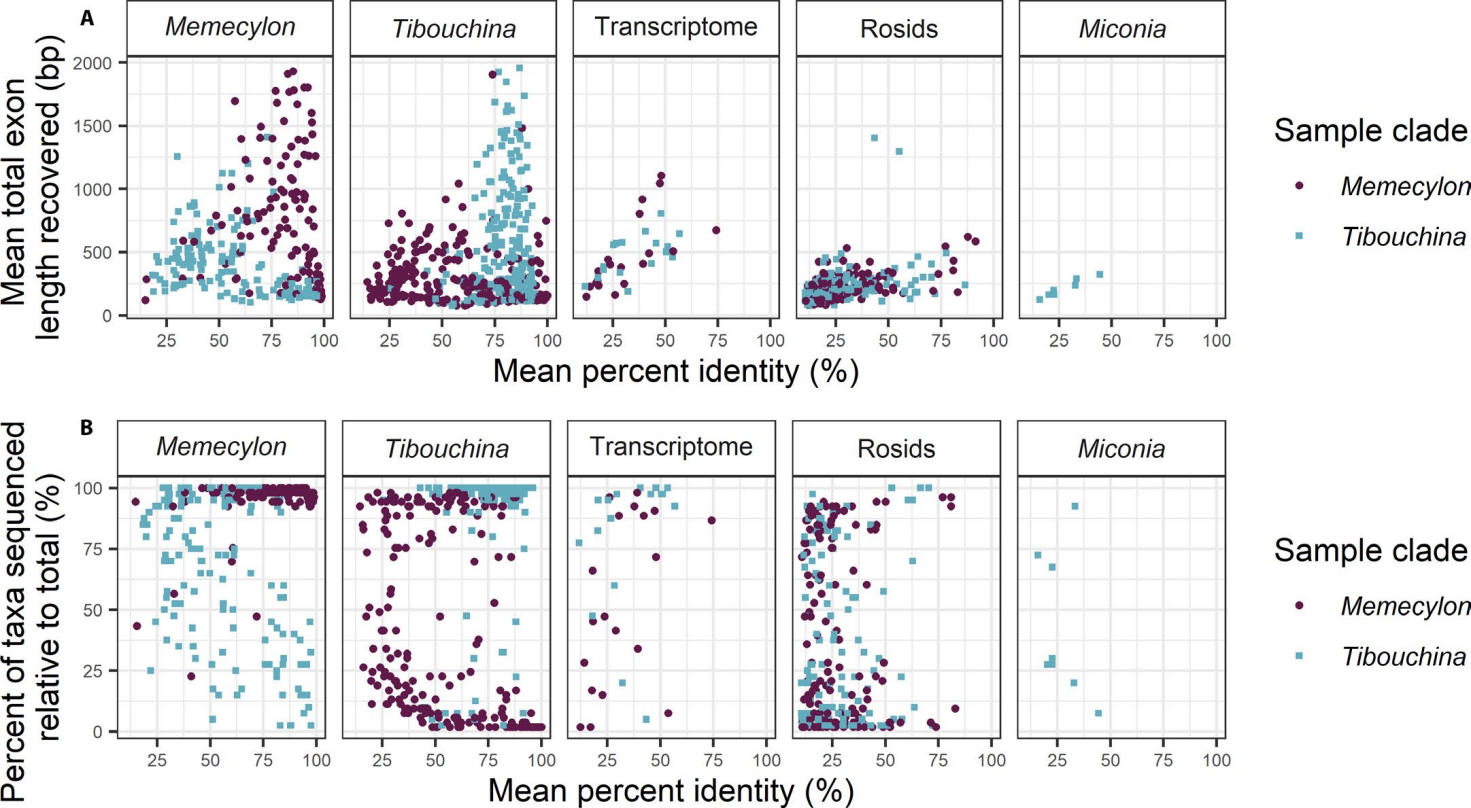
Percent identity of templates to recovered sequences are on the *x* axis with (A) mean total exon length shown on the *y* axis and (B) percent of taxa recovered for each template sequence shown on the *y* axis. Templates are grouped by the genomic source used for probe design for target enrichment of *Memecylon* (purple circles) and *Tibouchina* (blue squares).

Loci selected using MarkerMiner had higher success rates for each clade than the Angiosperms353 loci (Fig. 3). Probes that were designed from genome‐skimming data from the clade of interest were more likely to recover sequences for that clade. Templates with probes that were successful in capturing sequences from both clades had an average of 56.8% and 67.9% sequence identity with *Memecylon* species and *Tibouchina* species, respectively. Higher sequence identity was correlated with increased length of recovered sequences in both clades (Fig. 4A), especially for templates from sequences outside of the clades of interest. For templates developed from within the clade of interest, sequence identity was generally high (>75%), while sequencing success varied widely.

## DISCUSSION

We successfully targeted putatively SCN loci for two distantly related clades of interest (*Memecylon* and *Tibouchina* s.s.) within Melastomataceae, resulting in data sets of, on average, over 200 sequenced loci for each clade. We designed probes from 689 template sequences for 384 loci identified through our pipeline. We identified 193 loci that were recovered from more than 50% of species in each clade, and therefore putatively conserved between the two clades. We identified 56 template sequences from which probes were designed that may successfully target loci in both clades. Probes from these 56 templates will likely be successful in capturing sequences more broadly across Melastomataceae and these may serve as universal melastome loci. Many other loci were recovered for each individual clade, resulting in an average of approximately 648,000 bp (*Memecylon*) and 552,000 bp (*Tibouchina*) in total aligned length. This number of available base pairs represents a huge increase in the number of characters available for phylogenetic inference; we anticipate that this increase in data will substantially improve the resolution and support for phylogenetic relationships within these clades.

### Locus and probe comparisons

The 384 targeted loci were developed by four different methods, including 104 loci from MarkerMiner, 266 loci from the Angiosperms353 project, six functional loci, and eight previously published SCN loci. Our results showed differences in the success of the probes based on the method of locus selection. Loci that were selected using MarkerMiner were successful for both clades (Fig. 3). These loci were selected because of their presence in one or both transcriptomes within Melastomataceae, and because of their putatively single‐copy status according to the *A. thaliana* and/or *T. cacao* genomes. Because these loci were selected specifically for Melastomataceae, it was expected that they would be successfully sequenced from these two distantly related clades. However, gene loss or duplication within the clades of interest may affect the suitability of loci identified by these methods. Our results demonstrate that the loci identified from the *Miconia bicolor* and *Medinilla magnifica* transcriptomes are conserved across these disparate clades of Melastomataceae, in gene presence if not in gene copy number.

Although the loci identified by MarkerMiner are putatively single‐copy, high numbers of potential paralogs were identified within *Tibouchina*. Based on sequences recovered in our capture experiment, paralogy was less problematic within *Memecylon* (cf. paralog number, Table 2), indicating that paralogy issues in *Tibouchina* may be the result of recent genome or gene duplication events specific to the entire clade, or to members of that clade. However, intraspecific variation in the number of potential paralogs was observed within a subclade of *Memecylon* with population‐level sampling (Table 3). This variation could be an artifact of variable capture success, incorrect flagging of alleles as paralogs, or potentially the occurrence of within‐species gene duplications or losses. Ongoing work investigating ploidal levels in *Tibouchina* will identify the level of genome duplication in this clade and assess the utility of these potentially paralogous loci for phylogenetic analysis (J. Jantzen, unpublished data).

No methodology is guaranteed to identify and yield truly single‐copy loci in groups with few genomic resources, and assumptions are made during the locus selection process with respect to presumed absence of polyploids. Limitations to identifying SCN loci increase when genomic resources are limited, because although loci may be single‐copy in the reference taxon, depending on the evolutionary distance between the reference taxon and the target taxa, biological processes may have resulted in changes in gene copy number. More genomic resources from closely related taxa reduce the likelihood of unexpected changes in gene copy number. Paralogy is not easily avoided, although having a good understanding of ploidal variation in the taxa of interest may facilitate accurate selection of single‐copy loci.

The Angiosperms353 loci, using probes designed from various template sequences, were less successful than the MarkerMiner loci for these two clades, with higher variability in both sequence length recovered and number of taxa successfully targeted for each locus. We included a variety of template sequences assembled from the different genome‐skimming data sets for many of these universal loci. The genome source for the template affected the success of hybridization and sequencing, with higher success observed for those loci for which genome‐skim‐based probes were used and much lower success for probes designed from the more distantly related rosid sequences. However, for these Angiosperms353 loci, the genome‐skimming template sequences were typically much shorter than the overall length of the locus in the Angiosperms353 data set, resulting in shorter sequences recovered for the Angiosperms353 loci than expected. Similarly, when conducting the second tier of our pipeline, 190 of 266 loci included from the Angiosperms353 project did not recover contigs from the four genome‐skimming data sets, indicating that either the genome‐skimming process did not successfully sequence these low‐copy loci due to the shallow read depth, the loci were not assembled due to high sequence divergence, or, although unlikely, these loci may not be present in these taxa. Using amino acid sequences for assembly in HybPiper for probe design can improve success for assembly for highly divergent taxa, as we observed when comparing assembly for amino acid and nucleotide reference sequences of Angiosperms353 loci (Appendix S5). However, it appears that even when using the amino acid sequences for assembly, assembly success for the Angiosperms353 loci was still more variable than for the MarkerMiner loci, and that MarkerMiner sequences assembled were much longer overall. We found no correlation between read depth for each locus and the number of template sequences used (Appendix S6), with the exception of loci with only a single template, where this sequence was almost universally a distantly related rosid sequence. The slight decrease in read depth we observed for single‐template loci is likely due more to the high sequence divergence for those specific templates than to the number of templates.

For loci from all sources, as expected, probes designed based on genome‐skimming data from within the clade of interest were more successful in capturing and sequencing loci from taxa within those clades. For example, probes developed using genome‐skimming templates from *Memecylon* resulted in sequences that were longer and successfully captured loci from a larger number of species of *Memecylon* than the probes developed using *Tibouchina* genome‐skimming sequences, transcriptome sequences (from *Medinilla* or *Miconia*), or more distantly related rosids (Angiosperms353 probes). The converse is also true for probes designed from *Tibouchina* genome‐skimming templates with respect to success for taxa within *Tibouchina*. However, probes designed from 56 template sequences (for 37 unique loci) successfully captured loci in both distantly related clades, indicating that there is the potential to identify and target conserved loci across Melastomataceae. Because enrichment and sequencing were successful for both clades, including outgroup taxa (i.e., species of *Mouriri* and *Tibouchina* sensu lato), this set of loci shows potential for use in groups beyond those investigated in this study. Probes that can target sequences across distantly related clades will be a valuable resource for building sequence data sets for taxa from across Melastomataceae, potentially resolving uncertain relationships between major lineages.

Sequences that were captured and sequenced across a wide range of taxa, due in part to high sequence identity (Fig. 4B), may be useful for resolving higher‐order relationships within Melastomataceae because of the conserved nature of the sequences. However, sequences that are more divergent and have lower percent sequence identity are less likely to be captured and sequenced for a wide range of taxa, but are likely useful for resolving relationships between closely related taxa. Hence there is a tradeoff between sequence divergence and utility for resolving the phylogeny at the desired level, and being able to successfully target and sequence the loci across the taxa of interest. To assess the utility of these loci for resolving relationships at different scales, phylogenies must be reconstructed for each clade, both at the interspecific and intraspecific levels. Preliminary analyses in *Memecylon* at a broad phylogenetic scale find that these loci are able to successfully resolve interspecific relationships (P. Amarasinghe, unpublished data). Furthermore, preliminary population‐level analyses of the *Buxifolia* clade of *Memecylon* show that these loci provide insights into intraspecific relationships (P. Amarasinghe, unpublished data; see also Table 6).

**Table 6:** Variation statistics for alignments from the recovered sequences of *Memecylon* in interspecific and intraspecific phylogenetic analyses.

Statistics	Interspecific relationships									Intraspecific relationships								
	Exons			Introns			Supercontigs			Exons			Introns			Supercontigs		
	All	A353	MM	All	A353	MM	All	A353	MM	All	A353	MM	All	A353	MM	All	A353	MM
No. of genes	103	10	38	103	10	38	103	10	38	87	23	52	87	23	52	87	23	52
Alignment length	40,306	3381	25,922	26,109	4648	8998	79,170	8510	33,673	50,122	6483	40,735	42,087	14,253	23,377	123,983	26,447	66,082
Parsimony‐informative sites (%)	3819 (9.5)	278 (8.2)	2537 (9.8)	6812 (26.1)	1133 (24.4)	2819 (31.3)	11,182 (14.1)	1534 (18.0)	4536 (13.5)	777 (1.6)	86 (1.3)	651 (1.6)	2221 (5.3)	557 (3.9)	1540 (6.6)	3385 (2.7)	740 (2.8)	1797 (2.7)
Constant sites	30,471	2607	19,292	10,616	1894	3146	51,421	4492	21,175	47,826	6278	38,734	33,201	11,783	17,720	110,819	23,132	58,890
Missing data, %	4.2	2.1	0.7	5.6	3	2.4	3	0.2	1	0	0	0	0	0	0	1.9	0.9	0.6

Note

All = all selected loci for phylogenetic analysis; A353 = Angiosperms353 loci; MM = MarkerMiner loci.

The genome‐skimming data for two species each of *Memecylon* and Melastomateae facilitated probe design, resulting in well‐sampled phylogenomic data sets for both clades. This specificity in probe sequence, enabled by a few genomic resources, greatly increased the sequencing success for these loci. These results show the merit of custom‐designed probe sets (Kadlec et al., 2017), perhaps in combination with universal sets such as Angiosperms353. When it is possible to include them, taxon‐specific locus sets can also have higher success than universal locus sets (Chau et al., 2018). However, including loci from universal sets can increase the number of potential loci and enable combining different data sets using these shared loci (Chau et al., 2018), but we emphasize the importance of ensuring sufficiently low sequence divergence between probes and target sequences to allow for efficient capture. We also find that the taxon‐specific loci have higher proportions of variable sites than do universal loci (Table 6), indicating that conserved loci are less likely to be phylogenetically informative, although conserved loci also contain useful phylogenetic information (but see Chau et al., 2018).

### Comparison with similar methods

Diverse workflows similar to ours exist to customize target capture for different groups with few genomic resources; however, the approach presented here offers additional flexibility compared to prior studies by using low‐cost genome‐skimming data to generate clade‐specific probe sequences. Previous studies either took advantage of more extensive genomic resources (Mandel et al., 2014; Folk et al., 2015; Soto Gomez et al., 2019) or multiple closely related transcriptomes (Landis et al., 2016; Villaverde et al., 2018), including some from within the clade of interest (Crowl et al., 2017), to both identify loci and design probes. A second tier to obtain template sequences from the clade of interest is missing from the pipelines in these studies due to availability of transcriptome sequences within or more closely related to their focal clades. In our pipeline, genome‐skimming data is used to generate clade‐specific template sequences for target enrichment probe design. Although genome‐skimming data have been used in other studies (e.g., Weitemier et al., 2014; Vargas et al., 2019), those genome‐skimming reads were assembled for phylogenetic analysis directly, rather than for probe design. The second tier of our approach is novel and represents a step forward in target capture to make maximal use of genomic resources outside the clade of interest for locus identification and low‐cost options (i.e., genome skimming) for retrieving sequences from within the focal clade for probe design.

Several studies have used fewer resources to identify low‐copy nuclear loci for target enrichment (e.g., Weitemier et al., 2014; Schmickl et al., 2016). Although we used two genome‐skimming data sets for each clade of interest, two transcriptomes from the family, and two reference genomes outside of the family, our pipeline can be modified to use a single genome‐skimming data set and a single transcriptome if fewer genomic resources are available for other clades. To use our pipeline, at least one related transcriptome (e.g., within the family) is required for the first tier to identify putatively SCN loci; this is feasible for most angiosperms given the availability of phylogenetically representative transcriptomes from 1KP (Matasci et al., 2014; One Thousand Plant Transcriptomes Initiative, 2019). We recommend having at least one genome‐skimming data set or transcriptome within or closely related to the clade of interest for running the second tier to assemble taxon‐specific templates. Although having a closely related annotated genome is ideal, it is not necessary for this pipeline to work, as well‐annotated genomes for species including *A. thaliana* can be good references for even distantly related clades for target capture design.

### Caveats

In early studies using next‐generation sequencing, off‐target reads were readily obtained due to the inefficiency of the enrichment process. These reads generally included plastid sequences and other high‐copy loci including ribosomal DNA regions and some mitochondrial sequences. However, the specificity of target capture kits has improved, resulting in increased efficiency of target enrichment, and as reported here, very few off‐target reads (see also Singhal et al., 2017; de la Harpe et al., 2019). We therefore did not recover plastid genomes. To ensure that sequencing returns both on‐target enriched loci as well as desirable off‐target reads, enriched DNA samples should be combined with unenriched samples (Straub et al., 2012; Weitemier et al., 2014), or plastid probes can be designed alongside nuclear probes and included in the enrichment.

Although MarkerMiner aims to identify SCN loci, potential paralogs may be inadvertently included in the data set. Before proceeding to the second tier of the pipeline, a BLAST search of these loci against the reference transcriptomes could be conducted to identify any loci with multiple copies using a 90% sequence similarity threshold, as used by Schmickl et al. (2016). However, this process will not guarantee that the loci that are targeted are truly single‐copy in the study taxa.

In this study, when genome‐skimming reads were not successfully assembled for the MarkerMiner loci, these loci were discarded from the pipeline. However, we recommend including these loci if needed, using the transcriptome sequence as the template sequence. These loci could have been absent due to low coverage depths, and hence there is no direct evidence that they cannot be captured efficiently. Depending on the number of loci recovered through the pipeline and the cost associated, it may be worth including these loci in the set of templates for probe design.

Very few of the Angiosperms353 loci are represented by melastome sequences in the original probe set. When applying this pipeline in other clades with few genomic resources, if the clade of interest is not well‐represented in the probe set, we recommend optimizing the probe set using closely related sequences from genome‐skimming or transcriptomic data. We also recommend first recovering the appropriate transcripts from the alignments of the 1KP capstone paper (One Thousand Plant Transcriptomes Initiative, 2019) and using these sequences as references in the HybPiper step of Tier 2. This would increase the sequence similarity between the reference sequence and the genome‐skimming reads, potentially improving the assembly and recovery of contigs for these loci during the second tier of the pipeline and resulting in more loci for sequencing.

The laboratory procedures involved in target enrichment may also affect the recovery of loci. The company we employed (RAPiD Genomics) uses a proprietary workflow with optimized chemistry, and in internal lab comparisons, they find their library preparation method performs well compared to commercially available kits (A. Payton, RAPiD Genomics, Gainesville, Florida, USA, unpublished data). When conducting target capture, it is important to ensure that probe design (reflecting sequence divergence), probe construction, and library preparation are all optimized for the intended phylogenetic breadth of the study.

### Conclusions

We show that (1) genomic resources from taxa from outside the clade of interest can be effective in identifying putatively SCN loci, and (2) even with few genomic resources within a clade of interest, clade‐specific probes can be designed that improve the success of target enrichment and sequencing. Phylogeny reconstruction in Melastomataceae has until now relied on only a few loci, and relationships among major lineages remain poorly understood. We designed probes to sequence putatively SCN loci to resolve phylogenetic relationships across Melastomataceae, specifically within *Memecylon* and *Tibouchina*; these probes and loci will facilitate future studies of Melastomataceae and are available as a phylogenomic resource to the community. With these loci conserved in both *Memecylon* and *Tibouchina*, renewed efforts to resolve phylogenetic relationships among the highly diverse Melastomataceae should be successful. The methods outlined here can also be used to design probes to target SCN loci in other clades that lack many genomic resources.

## AUTHOR CONTRIBUTIONS

J.R.J., P.A., and R.A.F. conceived the ideas and designed the methodology. P.A., N.C., M.R., and F.A.M. provided the genome‐skimming data. J.R.J. and P.A. generated the sequence data, conducted the analyses, and led the writing of the manuscript. All authors contributed critically to the drafts and gave final approval for publication.

## Supporting information


**APPENDIX S1.** Accessions and voucher information for samples sequenced.

**APPENDIX S2.** DNA extraction protocol for silica gel–dried tissues of *Tibouchina* and *Memecylon* (Melastomataceae).

**APPENDIX S3.** Percent on‐target reads, read counts, read depth, the number of loci for which sequences were recovered, and the number of potential paralogs for all species.

**APPENDIX S4.** Locus‐ and template‐based statistics for sequencing success.

**APPENDIX S5.** Recovered sequence lengths for each category of template sequences.

**APPENDIX S6.** Read depth for template sequences.

## Data Availability

Cleaned reads have been deposited to the National Center for Biotechnology Information (NCBI) Sequence Read Archive (PRJNA592250, PRJNA573947, and PRJNA576018), template sequences and probe sequences are available on Dryad (https://doi.org/10.5061/dryad.8931zcrm2; Jantzen et al., 2020), and custom scripts are available at https://github.com/jjantzen/Probe_design. Accessions and voucher information for samples are listed in Appendix S1.
