# Supplementary material for: A two‐tier bioinformatic pipeline to develop probes for target capture of nuclear loci with applications in Melastomataceae
Source: Appl Plant Sci. 2020 May 9;8(5):e11345. doi: 10.1002/aps3.11345 (PMC7249273; doi:10.1002/aps3.11345)
Supplement: Supplementary file 1 — APPENDIX S1. Accessions and voucher information for samples sequenced. [file APS3-8-e11345-s001.docx]

**APPENDIX S1.** Accessions and voucher information for samples sequenced.

| Species | Herbarium | Barcode | Collection no. | Collector | Date of collection^a^ | Latitude | Longitude | NCBI accession |
| --- | --- | --- | --- | --- | --- | --- | --- | --- |
| *Memecylon afzelii* G. Don | MO | 6147058 | 947 | X.M. van der Burgt, J. Motoh, S. Njibili & M. Elangwe | 5/26/2007 | 5°01'N | 8°47"E | SAMN12920551 |
| *Memecylon amplexicaule* Roxb. | N/A | N/A | LSW44 | L.S. Wijedasa, M. Niisalo & T. Webb | 2/26/2012 | N/A | N/A | SAMN12920587 |
| *Memecylon australissimum* R. D. Stone & I. G. Mona | BNRH | 2838 | He2 | Stone & Amarasinghe | 12/15/2017 | 32°13'34.94"S | 28°53'49.68"E | SAMN12920571 |
| *Memecylon australissimum* R. D. Stone & I. G. Mona | NU | 2783 | H3 | Stone & Tenza | N/A | 31°81'91.96"S | 29°28'99.78"N | SAMN12920572 |
| *Memecylon bachmannii* Engler | NU | 2833 | C3 | Stone & Amarasinghe | 12/9/2017 | 31°24'04.43"S | 29°43'49.61"E | SAMN12920555 |
| *Memecylon bachmannii* Engler | NU | 2826 | E3 | Stone & Amarasinghe | 12/2/2017 | 29° 46'23.37"S | 30°48'21.63"E | SAMN12920564 |
| *Memecylon bachmannii* Engler | NU | 2826 | E5 | Stone & Amarasinghe | 12/2/2017 | 29° 46'23.33"S | 30°48'21.62"E | SAMN12920565 |
| *Memecylon bachmannii* Engler | NU | 2818 | G3 | Stone et al. | 11/23/2017 | 30°54'02.81"S | 30° 08'53.75"E | SAMN12920568 |
| *Memecylon bachmannii* Engler | NU | 2832 | GM5 | Stone & Amarasinghe | 12/9/2017 | 31°39'58.41''S | 29°86'88.11''N | SAMN12920569 |
| *Memecylon bachmannii* Engler | NU | 2835 | LP2 | Stone & Amarasinghe | 12/10/2017 | 31°40'86.97''S | 29°84'23.53"N | SAMN12920586 |
| *Memecylon bachmannii* Engler | NU | 2816 | EM4 | Stone & Amarasinghe | 11/22/2017 | 31°14'32.92"S | 30° 02'26.75"E | SAMN12920590 |
| *Memecylon bachmannii* Engler | NU | 2781 | MG1 | Stone, RD & Tenza | 6/10/2015 | 31°26’41.8”S | 29°38’26.9”E | SAMN12920592 |
| *Memecylon bachmannii* Engler | NU | 2837 | MK3 | Stone & Amarasinghe | 12/11/2017 | 31°19'03.72"S | 29°58'00.48"E | SAMN12920593 |
| *Memecylon bachmannii* Engler | NU | 2837 | MK6 | Stone & Amarasinghe | 12/11/2017 | 31°19'03.72"S | 29°58'00.48"E | SAMN12920594 |
| *Memecylon bachmannii* Engler | NU | 2814 | OM10 | Stone & Amarasinghe | 11/21/2017 | 30°72'36.32"S | 30°27'66.79"N | SAMN12920607 |
| *Memecylon bachmannii* Engler | NU | 2815 | OM12 | Stone & Amarasinghe | 11/21/2017 | 30°72'37.21"S | 30°27'64.12"E | SAMN12920608 |
| *Memecylon bachmannii* Engler | NU | 2785 | 2785 | Stone | 6/12/2015 | 31°38'56.7"S | 29°30'01.6"E | SAMN12920615 |
| *Memecylon bachmannii* Engler | NU | 2809 | U5 | Stone & Amarasinghe | 11/19/2017 | 31°02'37.92"S | 30°09'55.21"E | SAMN12920633 |
| *Memecylon caeruleum* Jack | SING | N/A | SIN-TA01 | P. Amarasinghe & D. Samanbaddha | 9/27/2019 | 1°21'46.76"N | 103°56'39.58"E | SAMN12920557 |
| *Memecylon calyptratum* K. Bremer | MO | 5010184 | 10626 | J.H. Beaman, R. S. Beaman, T. E. Beaman, E.A. Christensen & P. Decker | 7/9/1984 | 80.6°01.23'N | 116°41.01'E | SAMN12920556 |
| *Memecylon capitellatum* L. | FLAS | FLAS155002 | 50999 | F.R. Fosberg et al. | 1/4/1969 | 7° 42' 36.72'' N | 81° 41' 32.64'' E | SAMN12920567 |
| *Memecylon clarkeanum* Cogn. | PDA | N/A | K53 | P. Amarasinghe et al. | 7/14/2017 | 6°8'31.91"N | 80°12'6.73"E | SAMN12920578 |
| *Memecylon cuneatum* Thwaites | PDA | N/A | S10 | P. Amarasinghe et al. | 7/21/2017 | 6°29'1.35"N | 80°18'52.27"E | SAMN12920611 |
| *Memecylon discolor* Cogn. | PDA | N/A | Z008 | P. Amarasinghe et al. | 8/20/2017 | 6°25'21.23"N | 80°25'29.41"E | SAMN12920637 |
| *Memecylon excelsum* Blume | MO | 6448626 | 372 | T.J.F. Banguan, L.A.Farivo, R. Razalamalala, S. Al Lasut & A. Yani | 12/10/2012 | 00°40'01.43"N | 127°58'36.87"E | SAMN12920546 |
| *Memecylon flavescens* Gamble | JCB | N/A | srl10 | N. Page | N/A | 11° 18' 12.81'' N | 78° 20' 39.11'' E | SAMN12920623 |
| *Memecylon fruticosum* King | N/A | N/A | LSWT17 | L.S. Wijedasa | 5/14/2013 | N/A | N/A | SAMN12920588 |
| *Memecylon fuscescens* Thwaites | PDA | N/A | Z0010 | P. Amarasinghe et al. | 8/20/2017 | 6°27'57.22"N | 80°25'50.11"E | SAMN12920638 |
| *Memecylon giganteum* Alston | PDA | N/A | K51 | P. Amarasinghe et al. | 7/14/2017 | 6°15'56.53"N | 80°21'38.72"E | SAMN12920577 |
| *Memecylon gracillimum* Alston | PDA | N/A | D9 | P. Amarasinghe et al. | 7/19/2017 | 7°37'20.11"N | 80°24'36.74"E | SAMN12920561 |
| *Memecylon grande* Retz | PDA | N/A | Z004 | P. Amarasinghe et al. | 8/20/2017 | 6°25'16.95"N | 80°25'1.91"E | SAMN12920635 |
| *Memecylon hookeri* Thwaites | PDA | N/A | K46 | P. Amarasinghe et al. | 7/14/2017 | 6°14'43.86"N | 80°20'58.58"E | SAMN12920576 |
| *Memecylon kollimalayana* Viswanathan | N/A | N/A | srl14 | N. Page | N/A | 11° 59' 49.34'' N | 79° 49' 17.51'' E | SAMN12920626 |
| *Memecylon kosiense* R. D. Stone & I. G. Mona | NU | 2823 | K5 | Stone & Amarasinghe | 11/29/2017 | 27°03'76.84"S | 32°81'33.92"E | SAMN12920580 |
| *Memecylon lanceolatum* Blanco | US | 837376 | 975 | E. D. Merrill | 6/1/1916 | N/A | N/A | SAMN12920583 |
| *Memecylon lawsonii* Gamble | N/A | N/A | srl12 | N. Page | 3/17/2016 | N/A | N/A | SAMN12920624 |
| *Memecylon ligustrifolium* Champ. ex Benth. | FLAS | N/A | SHI XG 170701 | X. Shi | 7/1/2019 | 23° 30' 0'' N | 113° 15' 0'' E | SAMN12920585 |
| *Memecylon macrophyllum* Thwaites | PDA | N/A | Z006 | P. Amarasinghe et al. | 8/20/2017 | 6°22'53.92"N | 80°31'38.61"E | SAMN12920636 |
| *Memecylon malabaricum* Cogn. | MH | N/A | srl4 | N. Page | N/A | N/A | N/A | SAMN12920618 |
| *Memecylon maxwellii* Wijedasa | BKF | N/A | LSWT50 | L.S. Wijedasa et al. | 5/13/2013 | N/A | N/A | SAMN12920589 |
| *Memecylon natalense* Markgraf | BNRH | N/A | B2 | P. Amarasinghe et al. | 12/4/2017 | 25° 44'45.47"S | 31°18'45.14"E | SAMN12920552 |
| *Memecylon natalense* Markgraf | BNRH | N/A | B4 | P. Amarasinghe et al. | 12/4/2017 | 25° 44'45.44"S | 31°18'45.12"E | SAMN12920553 |
| *Memecylon natalense* Markgraf | NU | 2828 | BR1 | Stone & Amarasinghe | 12/2/2017 | 29° 46' 11.66'' S | 30° 50' 8.67'' E | SAMN12920554 |
| *Memecylon natalense* Markgraf | NU | 2830 | L1 | Stone & Amarasinghe | 12/9/2017 | 31°26'34.75"S | 29°43'55.92"E | SAMN12920581 |
| *Memecylon natalense* Markgraf | NU | 2830 | L3 | Stone & Amarasinghe | 12/9/2017 | 31°26'34.73"S | 29°43'55.98"E | SAMN12920582 |
| *Memecylon natalense* Markgraf | NU | 4877 | 4877 | Styles | 10/25/2014 | 29°11'15.21"S | 31°06'30.00"E | SAMN12920591 |
| *Memecylon natalense* Markgraf | NU | 2820 | MO2 | Stone & Amarasinghe | 11/24/2017 | 30°33'46.01"S | 30°38'82.32"E | SAMN12920595 |
| *Memecylon natalense* Markgraf | NU | 2821 | MO3 | Stone & Amarasinghe | 11/24/2017 | 30°33'47.45"S | 30°38'82.22"E | SAMN12920596 |
| *Memecylon natalense* Markgraf | NU | 2825 | N1 | Stone & Amarasinghe | 11/29/2017 | 28°44'50.96"S | 31°09'39.92"E | SAMN12920598 |
| *Memecylon natalense* Markgraf | NU | 2822 | O1 | Stone & Amarasinghe | 11/28/2017 | 28°49'37.01"S | 31° 44'35.24"E | SAMN12920600 |
| *Memecylon natalense* Markgraf | NU | 2822 | O4 | Stone & Amarasinghe | 11/28/2017 | 28°49'37.01"S | 31° 44'35.25"E | SAMN12920601 |
| *Memecylon natalense* Markgraf | NU | 2822 | O5 | Stone & Amarasinghe | 11/28/2017 | 28°49'37.02"S | 31° 44'35.21"E | SAMN12920602 |
| *Memecylon natalense* Markgraf | NU | 2822 | O7 | Stone & Amarasinghe | 11/28/2017 | 28°49'37.04"S | 31° 44'35.22"E | SAMN12920603 |
| *Memecylon natalense* Markgraf | NU | 2822 | O8 | Stone & Amarasinghe | 11/28/2017 | 28°49'37.03"S | 31° 44'35.22"E | SAMN12920604 |
| *Memecylon natalense* Markgraf | NU | 2810 | OH1 | Stone & Amarasinghe | 11/20/2017 | 30°43'01.41"S | 30° 15'28.96"E | SAMN12920605 |
| *Memecylon natalense* Markgraf | NU | 2812 | OM1 | Stone & Amarasinghe | 11/21/2017 | 30°70'74.82"S | 30°27'78.81"E | SAMN12920606 |
| *Memecylon natalense* Markgraf | NU | 2808 | S2 | Stone & Amarasinghe | 11/17/2017 | 29°54'13.45"S | 30° 56'31.01"E | SAMN12920612 |
| *Memecylon natalense* Markgraf | NU | 2808 | S7 | Stone & Amarasinghe | 11/17/2017 | 29°54'13.44"S | 30° 56'31.01"E | SAMN12920613 |
| *Memecylon natalense* Markgraf | NU | 2768 | 2768 | Stone et al. | 7/5/2013 | 27°49'40.31"S | 31°25'15.81''E | SAMN12920634 |
| *Memecylon orbiculare* Thwaites | PDA | N/A | Z0011 | P. Amarasinghe et al. | 8/20/2017 | 6°25'24.53"N | 80°25'36.61"E | SAMN12920639 |
| *Memecylon pauciflorum* Blume | NSW | NSW924277 | 1783 | D. L. Lewis | 5/13/2011 | 15°13'22.78"S | 129°38'53.84"E | SAMN12920548 |
| *Memecylon petiolatum* Alston | PDA | N/A | D11 | P. Amarasinghe et al. | 7/19/2017 | 7°36'43.48"N | 80°24'5.73"E | SAMN12920562 |
| *Memecylon procerum* Thwaites | PDA | N/A | K40 | P. Amarasinghe et al. | 7/14/2017 | 6°14'44.64"N | 80°20'54.02"E | SAMN12920574 |
| *Memecylon randeriana* S. M. Almeida & M. R. Almeida | N/A | N/A | srl20 | N. Page | 4/22/2018 | 19° 7' 55.62'' N | 73° 19' 28.21'' E | SAMN12920630 |
| *Memecylon rhinophyllum* Thwaites | PDA | N/A | D2 | P. Amarasinghe et al. | 7/19/2017 | 7°36'23.04"N | 80°24'46.13"E | SAMN12920558 |
| *Memecylon rivulare* K. Bremer | PDA | N/A | K11 | P. Amarasinghe et al. | 7/14/2017 | 6°8'36.62"N | 80°12'27.84"E | SAMN12920573 |
| *Memecylon rostratum* Thwaites | PDA | N/A | D6 | P. Amarasinghe et al. | 7/19/2017 | 7°36'51.33"N | 80°24'42.86"E | SAMN12920560 |
| *Memecylon schraderbergense* Mansf. | NSW | NSW893695 | 5797 | K. D. Q. Damas, K. M. Fazang & O.K. Paul | 3/8/2012 | 07°18'09.55" S | 147°07'50.67" E | SAMN12920550 |
| *Memecylon scolopacinum* Ridl. | MO | 3912702 | 8470 | John H. Beaman, R.S. Beaman, P. Decker & K. Medley | 2/4/1984 | 5°57'N | 116°34'W | SAMN12920614 |
| *Memecylon soutpansbergense* R. D. Stone & I. G. Mona | NU | 13706 | 13706 | Burrows et al. | 9/24/2013 | 22°38'55.44"S | 30°36'16.65"E | SAMN12920599 |
| *Memecylon* spD3 | PDA | N/A | D3 | P. Amarasinghe et al. | 7/19/2017 | 7°37'10.21"N | 80°24'31.73"E | SAMN12920559 |
| *Memecylon* spK45 | PDA | N/A | K45 | P. Amarasinghe et al. | 7/14/2017 | 6°14'44.65.75"N | 80°10'6.99.44"E | SAMN12920575 |
| *Memecylon* spN8 | PDA | N/A | N8 | P. Amarasinghe et al. | 7/14/2017 | 6°14'46.6.87"N | 80°20'52.82"E | SAMN12920597 |
| *Memecylon* spP3 | PDA | N/A | P3 | P. Amarasinghe et al. | 8/19/2017 | 6°43'53.55"N | 80°38'35.42"E | SAMN12920609 |
| *Memecylon* sp1 | N/A | N/A | srl1 | N/A | N/A | N/A | N/A | SAMN12920616 |
| *Memecylon* sp3 | N/A | N/A | srl3 | N/A | N/A | N/A | N/A | SAMN12920617 |
| *Memecylon* sp5 | FRLH | N/A | srl5 | N. Page | N/A | 11° 21' 4.56'' N | 76° 49' 53.24'' E | SAMN12920619 |
| *Memecylon* sp7 | FRLH | N/A | srl7 | N. Page | N/A | 8° 33' 54.52'' N | 77° 23' 18.36'' E | SAMN12920621 |
| *Memecylon* sp8 | N/A | N/A | srl8 | N. Page | N/A | N/A | N/A | SAMN12920622 |
| *Memecylon* sp16 | N/A | N/A | srl16 | N. Page | N/A | 12° 1' 53.79'' N | 77° 33' 57.36'' E | SAMN12920627 |
| *Memecylon* sp18 | FRLH | N/A | srl18 | N. Page | N/A | 11° 59' 49.31'' N | 79° 49' 17.55'' E | SAMN12920629 |
| *Memecylon* sp176 | MO | N/A | 176 | I. Gushilman, T. J. F. Banguan & I. Haris | 11/29/2012 | 00°32'34"N | 127°58'16"E | SAMN12920544 |
| *Memecylon* sp221 | MO | N/A | 221 | I. Gushilman, I. Haris, B. Sau, D. Loha & B. Eser | 12/8/2012 | 00°40'00"N | 127°58'27"E | SAMN12920545 |
| *Memecylon* sp501 | MO | N/A | 501 | I. Gushilman, B. Fabanyo, S. Lasut & R. Mahroji | 3/24/2013 | 00°31'34"N | 127°54'48"E | SAMN12920547 |
| *Memecylon* sp3471 | MO | 6427888 | 3471 | M. Merello, I. Gushilman, I. Haris, D. Loha & B. Eser | 12/6/2012 | 00°39'45'"N | 127°58'26"E | SAMN12920549 |
| *Memecylon sylvaticum* Thwaites | PDA | N/A | H6 | P. Amarasinghe et al. | 8/17/2017 | 7°16'52.5"N | 80°38'8.7"E | SAMN12920570 |
| *Memecylon symplociforme* Merr. | US | 1050204 | 15945 | A.D.E. Elmer | 5/1/1916 | 14°54'28.93"N | 120°44'39.12"E | SAMN12920631 |
| *Memecylon terminale* Dalz. | FRLH | N/A | srl6 | N. Page | N/A | 13° 9' 34.14'' N | 93° 0' 16.85'' E | SAMN12920620 |
| *Memecylon torricellense* Lauterb. | MO | 6373042 | 16241 | W. Takeuchi & D. Ama | 5/8/2002 | 6°40'S | 146°56'E | SAMN12920632 |
| *Memecylon umbellatum* Burm. | FLAS | FLAS155093 | 68091404 | R.G. Cooray & N. Wirawan | 9/14/1968 | 8° 24' 38.16''N | 80° 3' 3.60'' E | SAMN12920566 |
| *Memecylon umbellatum* Burm. | N/A | N/A | srl13 | N. Page | 4/16/2018 | 8° 32' 54.02'' N | 77° 18' 40.95'' E | SAMN12920625 |
| *Memecylon umbellatum* Burm. | N/A | N/A | srl17 | N. Page | N/A | 11° 36' 54.92'' N | 92° 37' 10.14'' E | SAMN12920628 |
| *Memecylon urceolatum* Cogn. | PDA | N/A | D15 | P. Amarasinghe et al. | 7/19/2017 | 7°36'43.42"N | 80°24'5.74"E | SAMN12920563 |
| *Memecylon varians* Thwaites | PDA | N/A | K56 | P. Amarasinghe et al. | 7/14/2017 | 6°8'32.42"N | 80°12'21.45"E | SAMN12920579 |
| *Memecylon wightii* Thwaites | PDA | N/A | L1 | P. Amarasinghe et al. | 7/13/2017 | 7°7'37.82"N | 80°42'38.71"E | SAMN12920584 |
| *Mouriri helleri* Britton | NYBG | 962763 | 2232 | J.D. Akerman | 2/2/1986 | 40°44'30.8"N | 73°59'21.5" | SAMN12920610 |
| *Tibouchina aegopogon* (Naudin) Cogn. | TBD | TBD | 45 | AL Freitas Oliveira et al | 1/9/2018 | 15˚48'04"S | 48˚48'52"W | SAMN12836987 |
| *Desmoscelis villosa* (Aubl.) Naudin | BHCB | BHCB 160936 | 865 | Rocha | 10/26/2012 | 3˚23'31"S | 61˚26'32"W | SAMN12837055 |
| *Tibouchina aegopogon* (Naudin) Cogn. | TBD | TBD | 56 | AL Freitas Oliveira et al | 1/9/2018 | 15˚46'54"S | 48˚49'15"W | SAMN12836989 |
| *Tibouchina aegopogon* (Naudin) Cogn. | TBD | TBD | 85 | AL Freitas Oliveira et al | 1/11/2018 | 14˚07'26"S | 47˚30'17"W | SAMN12836995 |
| *Tibouchina aegopogon* (Naudin) Cogn. | TBD | TBD | 91 | AL Freitas Oliveira et al | 1/12/2018 | 14˚08'03"S | 47˚40'29"W | SAMN12836996 |
| *Tibouchina aegopogon* (Naudin) Cogn. | TBD | TBD | 97 | AL Freitas Oliveira et al | 1/12/2018 | 14˚08'11"S | 47˚39'52"W | SAMN12836997 |
| *Tibouchina aegopogon* (Naudin) Cogn. | TBD | TBD | 107b | AL Freitas Oliveira et al | 1/14/2018 | 15˚44'26"S | 47˚58'38"W | SAMN12837000 |
| *Tibouchina aegopogon* (Naudin) Cogn. | TBD | TBD | 114 | AL Freitas Oliveira et al | 1/15/2018 | 16˚42'18"S | 47˚38'41"W | SAMN12837003 |
| *Tibouchina aegopogon* (Naudin) Cogn. | RB | 977028 | 5879 | Labiak, P. H. | 3/5/2015 | 11˚13'24"S | 47˚33'58"W | SAMN12837030 |
| *Tibouchina aegopogon* (Naudin) Cogn. | RB | RB 538333 | 295 | Drummond | 3/17/2012 | 18˚19'49"S | 48˚25'20"W | SAMN12837052 |
| *Tibouchina aegopogon* (Naudin) Cogn. | NY | 1164750 | 9523 | Almeda | 2/24/2009 | 15˚48'53"S | 48˚51'31"W | SAMN12837086 |
| *Tibouchina aegopogon* (Naudin) Cogn. | NY | 941543 | 34858 | Irwin et al | 1/23/1972 | 14˚28'17"S | 48˚33'32"W | SAMN12837127 |
| *Tibouchina aegopogon* (Naudin) Cogn. | NY | 748891 | 14453 | Irwin et al | 4/6/1966 | 14˚S | 46˚W | SAMN12837128 |
| *Tibouchina aegopogon* (Naudin) Cogn. | NY | 941530 | 11804 | Irwin et al | 1/19/1966 | 14˚S | 46˚W | SAMN12837129 |
| *Tibouchina aegopogon* (Naudin) Cogn. subsp. *angustifolia* Cogn. | TBD | TBD | 83 | AL Freitas Oliveira et al | 1/11/2018 | 13˚55'51"S | 47˚25'56"W | SAMN12836994 |
| *Tibouchina aegopogon* (Naudin) Cogn. subsp. *angustifolia* Cogn. | TBD | TBD | 103 | AL Freitas Oliveira et al | 1/13/2018 | 13˚55'48"S | 47˚25'59"W | SAMN12836998 |
| *Tibouchina aegopogon* (Naudin) Cogn. subsp. *angustifolia* Cogn. | UEC | UEC 188147 | 1199 | Meyer, F. S. | 3/22/2012 | 14˚5'43"S | 47˚29'34"W | SAMN12837038 |
| *Tibouchina aegopogon* (Naudin) Cogn. subsp. *angustifolia* Cogn. | NY | 3257186 | 5891 | Labiak et al | 3/6/2015 | 11˚41'39"S | 47˚42'3"W | SAMN12837113 |
| *Tibouchina aegopogon* (Naudin) Cogn. subsp. *angustifolia* Cogn. | NY | none | 5689 | Thomas et al | 12/4/1987 | 13˚59'S | 60˚43'W | SAMN12837114 |
| *Tibouchina aegopogon* aff. | TBD | TBD | 125 | AL Freitas Oliveira et al | 1/22/2018 | 15˚24'49"S | 55˚50'37"W | SAMN12837008 |
| *Tibouchina aegopogon* aff. | TBD | TBD | 130 | AL Freitas Oliveira et al | 1/23/2018 | 15˚26'14"S | 55˚50'10"W | SAMN12837010 |
| *Tibouchina albescens* Cogn. ex P. J. F. Guim., A. L. F. Oliveira & R. Romero | TBD | TBD | 107a | AL Freitas Oliveira et al | 1/13/2018 | 14˚10'17"S | 47˚31'17"W | SAMN12836999 |
| *Tibouchina albescens* Cogn. ex P. J. F. Guim., A. L. F. Oliveira & R. Romero | RB | RB 538353 | 342 | Drummond | 3/22/2012 | 14˚17'49"S | 47˚50'19"W | SAMN12837048 |
| *Tibouchina albescens* Cogn. ex P. J. F. Guim., A. L. F. Oliveira & R. Romero | RB | RB 538345 | 331 | Drummond | 3/21/2012 | 14˚11'23"S | 47˚46'28"W | SAMN12837051 |
| *Tibouchina albescens* Cogn. ex P. J. F. Guim., A. L. F. Oliveira & R. Romero | NY | 941564 | 21664 | Irwin et al | 3/23/1968 | 16˚21'26"S | 48˚58'55"W | SAMN12837115 |
| *Tibouchina aspera* Aubl. | RB | RB 589217 | 815 | Bigio | 5/7/2013 | 9˚39'11"S | 65˚10'17"W | SAMN12837031 |
| *Tibouchina aspera* Aubl. | RB | RB 564102 | 877 | Rocha & Guimarães | 10/28/2012 | 4˚29'32"S | 61˚9'51"W | SAMN12837032 |
| *Tibouchina aspera* Aubl. | MBM | MBM 203279 | 65628 | Hatschbach, G. et al. | 11/16/1996 | 15˚0'29"S | 59˚57'2"W | SAMN12837034 |
| *Tibouchina aspera* Aubl. | UEC | UEC 138639 | 944 | Costa, S. M. & Barbosa, T. D. M. | 3/23/2011 | 1˚1'33"N | 61˚8'45"W | SAMN12837039 |
| *Tibouchina aspera* Aubl. | UEC | UEC 125996 | 673 | Alencar, M. E. | 6/27/1999 | 4˚2'60"S | 41˚26'24"W | SAMN12837040 |
| *Tibouchina aspera* Aubl. | BHCB | BHCB 160931 | 860 | Rocha | 10/25/2012 | 3˚20'18"S | 61˚24'15"W | SAMN12837046 |
| *Tibouchina aspera* Aubl. | NY | 1462596 | 512 | Torke et al | 5/26/2009 | 16˚29'38"S | 68˚7'9"W | SAMN12837087 |
| *Tibouchina aspera* Aubl. | NY | 2571882 | 452 | Gorts - van Rijn et al | 11/28/1992 | 6˚30'N | 58˚23'W | SAMN12837088 |
| *Tibouchina aspera* Aubl. | NY | 2103852 | 2033 | Michelangeli | 8/12/2013 | 3˚39'27"N | 56˚12'13"W | SAMN12837089 |
| *Tibouchina aspera* Aubl. | NY | 1654094 | 409 | Martin | s.d. | 3˚56'9"N | 53˚7'33"W | SAMN12837096 |
| *Tibouchina aspera* Aubl. | NY | 1653298 | 423 | Martin | 11/10/2004 | 3˚56'9"N | 53˚7'33"W | SAMN12837097 |
| *Tibouchina aspera* Aubl. | NY | 2571935 | 30998 | Maguire, Cowan, Wurdack | 1/17/1951 | 7˚19'2"N | 63˚22'26" | SAMN12837099 |
| *Tibouchina aspera* Aubl. | NY | 2571925 | 41465 | Maguire, Wurdack, Keith | 9/15/1957 | 9˚0'8"N | 71˚54'38"W | SAMN12837100 |
| *Tibouchina aspera* Aubl. | NY | 2571953 | 9368 | Bunting | 7/22/1980 | 10˚25'3"N | 71˚1'0"W | SAMN12837101 |
| *Tibouchina aspera* Aubl. | NY | 2571945 | 54087 | Maguire et al | 7/8/1963 | 3˚26'17"N | 57˚13'56"W | SAMN12837102 |
| *Tibouchina aspera* Aubl. | NY | 1290931 | 10957 | Anderson | 2/14/1974 | 7˚30'S | 57˚15'W | SAMN12837103 |
| *Tibouchina aspera* Aubl. | NY | 1856150 | 775 | Rocha, Cabral | 10/5/2012 | 1˚28'44"S | 60˚58'08"W | SAMN12837104 |
| *Tibouchina aspera* Aubl. | NY | 1290914 | 34736 | Nee | 4/10/1987 | 10˚44'S | 65˚15'W | SAMN12837105 |
| *Tibouchina aspera* Aubl. | NY | 2571894 | 1666 | Agostini, Agostini | 4/19/1973 | 8˚49'N | 62˚27'W | SAMN12837106 |
| *Tibouchina aspera* Aubl. | NY | 1290940 | 1143 | Nelson | 4/23/1981 | 2˚26'56"S | 54˚42'3"W | SAMN12837107 |
| *Tibouchina aspera* Aubl. | NY | 2571903 | 2350 | Christenhusz | 2/14/2003 | 4˚47'N | 52˚26'W | SAMN12837108 |
| *Tibouchina aspera* Aubl. | NY | 1290927 | 7585 | Daly et al | 3/22/1992 | 8˚16'S | 72˚47'W | SAMN12837109 |
| *Tibouchina aspera* Aubl. | NY | 1290921 | 30.04 | Prance et al | 4/21/1986 | 3˚2'S | 60˚35'W | SAMN12837110 |
| *Tibouchina aspera* Aubl. | NY | 2529709 | 2827 | Fryxell | 11/23/1976 | 15˚53'N | 84˚42'W | SAMN12837111 |
| *Tibouchina barbigera* aff. | TBD | TBD | 132 | AL Freitas Oliveira et al | 1/23/2018 | 15˚26'13"S | 55˚50'10"W | SAMN12837011 |
| *Tibouchina barbigera* Baill. | TBD | TBD | 60 | AL Freitas Oliveira et al | 1/9/2018 | 15˚47'52"S | 48˚48'60"W | SAMN12836990 |
| *Tibouchina barbigera* Baill. | TBD | TBD | 135 | AL Freitas Oliveira et al | 1/24/2018 | 14˚18'39"S | 52˚10'08"W | SAMN12837013 |
| *Tibouchina barbigera* Baill. | NY | 941601 | 2085 | Dubs | 3/16/1996 | 15˚26'S | 55˚44'W | SAMN12837121 |
| *Tibouchina barbigera* Baill. | NY | 03909667 | 1141 | Balcazar et al | 5/3/1997 | 17˚14'54"S | 58˚39'07"W | SAMN12837122 |
| *Tibouchina barbigera* Baill. | NY | 941580 | 17761 | Irwin et al | 6/26/1966 | 16˚57'11"S | 51˚48'33"W | SAMN12837123 |
| *Tibouchina barbigera* Baill. | NY | 941599 | 117 | Brooks et al | 4/18/1988 | 14˚51'S | 48˚52'W | SAMN12837124 |
| *Tibouchina barbigera* Baill. | NY | 880251 | 2097 | Romero et al | 4/7/1995 | 18˚54'41"S | 48˚16'32"W | SAMN12837125 |
| *Tibouchina barbigera* Baill. | NY | 941584 | 1524 | Ratter et al | 5/25/1968 | 12˚54'S | 51˚52'W | SAMN12837126 |
| *Tibouchina barbigera* Baill. cf *araguaiensis* | NY | 1290958 | 8798 | Plowman et al | 2/13/1980 | 8˚03'S | 50˚10'W | SAMN12837130 |
| *Tibouchina barnebyana* Wurdack | BHCB | BHCB 154431 | 290 | Rocha | 2/10/2012 | 13˚0'10"S | 41˚20'50"W | SAMN12837057 |
| *Tibouchina bipenicellata* (Naudin) Cogn. | NY | 3375124 | 39837 | Wurdack, Monachino | 12/12/1955 | 6˚2'12"N | 67˚23'6"W | SAMN12837082 |
| *Tibouchina bipenicellata* (Naudin) Cogn. | NY | 3375132 | 44055 | Maguire, Maguire | 8/30/1959 | 4˚9'9"N | 73˚38'16"W | SAMN12837083 |
| *Tibouchina bipenicellata* (Naudin) Cogn. | NY | 3375105 | 1335 | Burch et al | 12/25/1966 | 7˚46'14"N | 80˚43'17"W | SAMN12837084 |
| *Tibouchina bipenicellata* (Naudin) Cogn. | NY | 3375096 | 8002 | Santamaria et al | 10/10/2009 | 9˚53'33.5"N | 84˚29'0.7"W | SAMN12837085 |
| *Tibouchina bipenicellata* (Naudin) Cogn. | NY | 1116598 | 683 | Michelangeli | 1/16/2001 | 7˚54'55"˚N | 72˚8'30"W | SAMN12837094 |
| *Tibouchina bipenicellata* (Naudin) Cogn. | NY | 1101014 | 735 | Michelangeli | 12/9/2001 | 10˚9'31"N | 69˚51'35"W | SAMN12837095 |
| *Tibouchina bruniana* P. J. F. Guim. | RB | 242767 | 2637 | R Marquete, A Vaz, RC Mendonca, ML Fonseca & GN Jesus | 10/18/1996 | 14˚28'26"S | 48˚27'35"W | SAMN12837026 |
| *Tibouchina bruniana* P. J. F. Guim. | UEC | UEC 69933 | 3070 | Filgueiras, T. S. et al. | 11/8/1994 | 14˚28'26"S | 48˚27'35"W | SAMN12837041 |
| *Tibouchina bruniana* P. J. F. Guim. | UEC | UEC 112154 | 3533 | Filgueiras, T. S. et al. | 4/1/1999 | 14˚28'26"S | 48˚27'35"W | SAMN12837042 |
| *Tibouchina catharinae* Pittier | NY | 3375142 | 28463 | Maguire, Politi | 1/20/1949 | 4˚57'54"N | 67˚19'50"W | SAMN12837064 |
| *Tibouchina catharinae* Pittier | NY | 3375141 | 28263 | Maguire, Politi | 1/10/1949 | 5˚57'54"N | 67˚19'50"W | SAMN12837065 |
| *Tibouchina cf albescens* Cogn. ex P. J. F. Guim., A. L. F. Oliveira & R. Romero | TBD | TBD | 134 | AL Freitas Oliveira et al | 1/24/2018 | 14˚18'39"S | 52˚10'08"W | SAMN12837012 |
| *Tibouchina dissitiflora* Wurdack | NY | 2572113 | 42268 | Maguire, Wurdack, Maguire | 12/2/1957 | 0˚56'27"N | 65˚54'31"W | SAMN12837060 |
| *Tibouchina dissitiflora* Wurdack | NY | 2572107 | 10933 | Huber | 10/20/1985 | 5˚55'N | 66˚26'W | SAMN12837112 |
| *Tibouchina dubia* Cogn. | TBD | TBD | 421 | Guimaraes | TBD | TBD | TBD | SAMN12837056 |
| *Tibouchina duidae* Gleason | NY | 2572122 | 30226 | Maguire, Cowan, Wurdack | 12/14/1950 | 3˚51'12"N | 65˚45'46"W | SAMN12837062 |
| *Tibouchina duidae* Gleason | NY | 2572125 | 31169 | Cowan, Wurdack | 2/2/1951 | 4˚24'60"N | 65˚49'59"W | SAMN12837063 |
| *Tibouchina edmundoi* Brade | TBD | TBD | 854 | L.V. Vasconcelos & R. Jaffé | 5/21/2016 | 6˚27'47"S | 50˚52'30"W | SAMN12837044 |
| *Tibouchina edmundoi* Brade | NY | 1290965 | 6253 | Pires et al | 12/14/1956 | 10˚11'17"S | 54˚27'49"W | SAMN12837072 |
| *Tibouchina edmundoi* Brade | NY | 1290967 | 158 | Secco et al | 5/14/1982 | 5˚35'30"S | 50˚10'60"W | SAMN12837073 |
| *Tibouchina exasperata* (Naudin) Cogn. | UEC | UEC 11042 | 7331 | Benson, W. W. & Morais, H. C. | 12/26/1977 | 19˚35'36"S | 46˚56'26"W | SAMN12837043 |
| *Tibouchina fraterna* N. E. Br. | UPCB | 79660 | 1361 | Reginato, M, Beltrame, MH | 2/1/2013 | 11˚42'35"S | 47˚43'24"W | SAMN12837028 |
| *Tibouchina fraterna* N. E. Br. | NY | 1873820 | 8248 | Forzza | 11/8/2014 | 4˚35'50"N | 60˚9'45"W | SAMN12837090 |
| *Tibouchina fraterna* N. E. Br. | NY | 1100829 | 512 | Michelangeli | 2/5/2000 | 5˚58'8"N | 62˚32'22"W | SAMN12837093 |
| *Tibouchina gracilis* (Bonpl.) Cogn. | TBD | TBD | 117 | AL Freitas Oliveira et al | 1/17/2018 | 19˚10'11"S | 48˚23'25"W | SAMN12837004 |
| *Tibouchina inopinata* Wurdack | MBM | MBM 142775 | 2988 | Almeda F. et al. | 7/9/1977 | 9˚51'49"N | 83˚54'58"W | SAMN12837036 |
| *Tibouchina johnwurdackiana* Todzia | RB | 695504 | 306 | Drummond | 3/18/2012 | 16˚07'22"S | 50˚18'320"W | SAMN12837027 |
| *Tibouchina johnwurdackiana* Todzia | RB | RB 538335 | 306 | Drummond | 3/18/2012 | 16˚43'60"S | 50˚23'20"W | SAMN12837045 |
| *Tibouchina johnwurdackiana* Todzia | RB | RB 538337 | 311 | Drummond | 3/18/2012 | 16˚22'0"S | 51˚21'59"W | SAMN12837050 |
| *Tibouchina karstenii* Cogn. | NY | 2594262 | 4780 | Luteyn et al | 1/10/1976 | 3˚7'46"N | 73˚45'9"W | SAMN12837069 |
| *Tibouchina karstenii* Cogn. | NY | 2594261 | 7659 | Cuatrecasas | 11/11/1939 | 2˚35'3"N | 72˚38'54"W | SAMN12837070 |
| *Tibouchina kunhardtii* Gleason | NY | 2594268 | 27794A | Maguire, Politi | 12/20/1948 | 4˚57'53"N | 67˚1950"W | SAMN12837066 |
| *Tibouchina llanorum* Wurdack | NY | 2594472 | 4881 | Aristeguieta | Sep-62 | 9˚22'60"N | 68˚20'2"W | SAMN12837067 |
| *Tibouchina llanorum* Wurdack | NY | 2594477 | 5393 | Killeen et al | 4/20/1993 | 14˚32'0"S | 61˚1'00"W | SAMN12837068 |
| *Tibouchina llanorum* Wurdack | NY | 1117498 | 348 | Michelangeli | 6/27/1996 | 5˚36'43"N | 66˚7'6"W | SAMN12837091 |
| *Tibouchina llanorum* Wurdack | VEN | none | 736 | Michelangeli | unknown | unknown | unknown | SAMN12837098 |
| *Tibouchina mathaei* Cogn. | NY | 2594730 | 5216 | Soukup | 1/17/1965 | 6˚4'0"N | 77˚8'30"W | SAMN12837071 |
| *Tibouchina melastomoides* (Naudin) Cogn. | TBD | TBD | 93 | AL Freitas Oliveira et al | 1/12/2018 | 14˚07'42"S | 47˚˚40'19"W | SAMN12837029 |
| *Tibouchina melastomoides* (Naudin) Cogn. | RB | RB 613831 | 605 | Versiane | 5/22/2013 | 15˚45'38"S | 48˚55'8"W | SAMN12837033 |
| *Tibouchina melastomoides* (Naudin) Cogn. | RB | RB 539204 | 322 | Drummond | 3/20/2012 | 14˚10'47"S | 47˚50'32"W | SAMN12837047 |
| *Tibouchina melastomoides* (Naudin) Cogn. | TBD | TBD | 329 | Drummond | 3/21/2012 | TBD | TBD | SAMN12837054 |
| *Tibouchina nigricans* Cogn. ex P. J. F. Guim., A. L. F. Oliveira & R. Romero | TBD | TBD | 67 | AL Freitas Oliveira et al | 1/10/2018 | 15˚49'23"S | 48˚42'08"W | SAMN12836991 |
| *Tibouchina nigricans* Cogn. ex P. J. F. Guim., A. L. F. Oliveira & R. Romero | TBD | TBD | 71 | AL Freitas Oliveira et al | 1/10/2018 | 15˚49'22"S | 48˚42'07"W | SAMN12836992 |
| *Tibouchina nigricans* Cogn. ex P. J. F. Guim., A. L. F. Oliveira & R. Romero | TBD | TBD | 110 | AL Freitas Oliveira et al | 1/15/2018 | 15˚39'50"S | 47˚56'13"W | SAMN12837001 |
| *Tibouchina nigricans* Cogn. ex P. J. F. Guim., A. L. F. Oliveira & R. Romero | TBD | TBD | 112 | AL Freitas Oliveira et al | 1/15/2018 | 15˚39'50"S | 47˚56'13"W | SAMN12837002 |
| *Tibouchina nigricans* Cogn. ex P. J. F. Guim., A. L. F. Oliveira & R. Romero | HUFU | 71372 | 457 | Versiane, AFA; Pacheco, RA | 12/13/2012 | 15˚49'30"S | 48˚41'36"W | SAMN12837059 |
| *Tibouchina papyrifera* (Pohl ex Naudin) Cogn. | NY | 941758 | 2242 | Mello-silva et al | 11/29/2003 | 16˚0'5"S | 50˚3'5"W | SAMN12837116 |
| *Tibouchina papyrus* (Pohl) Toledo | TBD | TBD | 53 | AL Freitas Oliveira et al | 1/9/2018 | 15˚48'07"S | 48˚51'13"W | SAMN12836988 |
| *Tibouchina papyrus* (Pohl) Toledo | TBD | TBD | 304 | Drummond | TBD | TBD | TBD | SAMN12837053 |
| *Tibouchina papyrus* (Pohl) Toledo | NY | 2332269 | 1106 | Miranda | 5/3/2008 | 16˚7'35"S | 50˚12'51"W | SAMN12837117 |
| *Tibouchina pogonanthera* (Naudin) Cogn. | MBM | MBM 381894 / ESA 44313 | 14007 | Souza, V.C. et al. | 3/14/1997 | 11˚20'33"S | 50˚35'24"W | SAMN12837035 |
| *Tibouchina ramboi* Brade | TBD | TBD | 441 | Guimaraes | TBD | TBD | TBD | SAMN12837058 |
| *Tibouchina rosanae* P. J. F. Guim. & Woodgyer | INPA | 111743 | 1404 | Teixeira, Ramos, Silva, Mota, Freitas and Bilby | 9/2/1983 | 8˚45'39.44"S | 63˚54'1.59"W | SAMN12837025 |
| *Tibouchina rosanae* P. J. F. Guim. & Woodgyer | UPCB | UPCB 78689 | 3217 | Soares, C. R. A. et al. | 3/10/2011 | 9˚45'22"S | 52˚38'6"W | SAMN12837037 |
| *Tibouchina sipapoana* Gleason | NY | 3530063 | 28466 | Maguire, Politi | 1/20/1949 | 4˚57'53"N | 67˚1950"W | SAMN12837061 |
| *Tibouchina* sp. nov*.* 3 | TBD | TBD | 123 | AL Freitas Oliveira et al | 1/22/2018 | 15˚24'49"S | 55˚50'37"W | SAMN12837007 |
| *Tibouchina* sp. nov. 3 | TBD | TBD | 128 | AL Freitas Oliveira et al | 1/22/2018 | 15˚23'34"S | 55˚50'05"W | SAMN12837009 |
| *Tibouchina* sp. nov. 5 | TBD | TBD | 151 | AL Freitas Oliveira et al | 1/26/2018 | 09˚56'15"S | 52˚24'23"W | SAMN12837021 |
| *Tibouchina* sp. nov. 5 | TBD | TBD | 153 | AL Freitas Oliveira et al | 1/26/2018 | 09˚56'15"S | 52˚24'22"W | SAMN12837022 |
| *Tibouchina* sp. nov. 5 | TBD | TBD | 154 | AL Freitas Oliveira et al | 1/26/2018 | 09˚56'14"S | 52˚24'22"W | SAMN12837023 |
| *Tibouchina* sp. nov. 6 | TBD | TBD | 144 | AL Freitas Oliveira et al | 1/25/2018 | 09˚48'18"S | 52˚26'34"W | SAMN12837017 |
| *Tibouchina* sp. nov. 6 | TBD | TBD | 150 | AL Freitas Oliveira et al | 1/25/2018 | 09˚55'24"S | 52˚24'21"W | SAMN12837020 |
| *Tibouchina* sp. nov. 6 | TBD | TBD | 156 | AL Freitas Oliveira et al | 1/27/2018 | 11˚01'16"S | 51˚38'38"W | SAMN12837024 |
| *Tibouchina* sp. nov. 7 | TBD | TBD | 142 | AL Freitas Oliveira et al | 1/25/2018 | 09˚48'13"S | 52˚26'30"W | SAMN12837016 |
| *Tibouchina* sp. nov. 7 | TBD | TBD | 147 | AL Freitas Oliveira et al | 1/25/2018 | 09˚48'10"S | 52˚26'09"W | SAMN12837018 |
| *Tibouchina* sp. nov. 8 | TBD | TBD | 119 | AL Freitas Oliveira et al | 1/22/2018 | 15˚24'39"S | 55˚50'11"W | SAMN12837005 |
| *Tibouchina* sp. nov. 8 | TBD | TBD | 120 | AL Freitas Oliveira et al | 1/22/2018 | 15˚24'39"S | 55˚50'12"W | SAMN12837006 |
| *Tibouchina* sp. nov. 9 | TBD | TBD | 140 | AL Freitas Oliveira et al | 1/25/2018 | 09˚48'13"S | 52˚26'30"W | SAMN12837015 |
| *Tibouchina* sp. nov. 9 | TBD | TBD | 149 | AL Freitas Oliveira et al | 1/25/2018 | 09˚45'10"S | 52˚18'32"W | SAMN12837019 |
| *Tibouchina spruceana* Cogn. | NY | 2595455 | 34592 | Nee | 3/31/1987 | 12˚30'S | 64˚08'W | SAMN12837075 |
| *Tibouchina spruceana* Cogn. | NY | 1290987 | 1448 | Rose, Cordeiro | 2/12/1977 | 2˚49'28"N | 60˚40'33"W | SAMN12837076 |
| *Tibouchina spruceana* Cogn. | NY | 2332293 | 6518 | Richards | 7/25/1968 | 10˚55'12"S | 50˚10'60"W | SAMN12837077 |
| *Tibouchina spruceana* Cogn. | NY | 941795 | 1 | Valle, Valio | Sep-72 | 12˚55'6"S | 53˚57'39"W | SAMN12837078 |
| *Tibouchina spruceana* Cogn. | NY | 1290983 | 29933 | Prance et al | 4/20/1986 | 3˚2'60"S | 60˚36'0"W | SAMN12837079 |
| *Tibouchina spruceana* Cogn. | NY | 2595461 | 36176 | Maguire, Wurdack, Bunting | 11/12/1953 | 5˚39'44"N | 67˚34'58"W | SAMN12837080 |
| *Tibouchina spruceana* Cogn. | NY | 2595460 | 34703 | Maguire, Wurdack | 3/25/1953 | 3˚9'59"N | 65˚33'5"W | SAMN12837081 |
| *Tibouchina striphnocalyx* (DC.) Gleason | NY | 1164323 | 379 | Michelangeli | 1/4/1997 | 1˚55'15"N | 67˚3'19"W | SAMN12837092 |
| *Tibouchina tuberosa* Cogn. | NY | 941853 | 9827 | Anderson | 5/6/1973 | 15˚53'24"S | 52˚15'24"W | SAMN12837074 |
| *Tibouchina verticillaris* Cogn. | TBD | TBD | 78 | AL Freitas Oliveira et al | 1/11/2018 | 13˚55'41"S | 47˚25'56"W | SAMN12836993 |
| *Tibouchina verticillaris* Cogn. | TBD | TBD | 138 | AL Freitas Oliveira et al | 1/24/2018 | 13˚36'02"S | 51˚56'06"W | SAMN12837014 |
| *Tibouchina verticillaris* Cogn. | RB | RB 538342 | 309 | Drummond | 3/18/2012 | 16˚22'0"S | 51˚21'59"W | SAMN12837049 |
| *Tibouchina verticillaris* Cogn. | NY | 941866 | 25340 | Irwin et al | 1/25/1970 | 18˚9'52"S | 47˚56'42"W | SAMN12837118 |
| *Tibouchina verticillaris* Cogn. | NY | 748837 | 34591 | Irwin et al | 1/19/1972 | 15˚51'5"S | 48˚57'31"W | SAMN12837119 |
| *Tibouchina verticillaris* Cogn. | NY | 1019604 | 1859 | Sasaki | 1/25/2008 | 9˚40'2.3"S | 55˚12'55.9"W | SAMN12837120 |

*Note:* NCBI = National Center for Biotechnology Information.

^a^Collection date is presented as month, day, year.
