# Supplementary material for: A two‐tier bioinformatic pipeline to develop probes for target capture of nuclear loci with applications in Melastomataceae
Source: Appl Plant Sci. 2020 May 9;8(5):e11345. doi: 10.1002/aps3.11345 (PMC7249273; doi:10.1002/aps3.11345)
Supplement: Supplementary file 2 — APPENDIX S2. DNA extraction protocol for silica gel–dried tissues of Tibouchina and Memecylon (Melastomataceae). [file APS3-8-e11345-s002.docx]

RNase A

**Appendix S2.** DNA extraction protocol for silica gel–dried tissues of *Tibouchina* and *Memecylon* (Melastomataceae).

Modified from: Soltis lab protocol, 2002, which is based on Doyle and Doyle (1987) and Cullings (1992)

**Materials**

1.5 mL Eppendorf tubes

Zirconia beads

2× CTAB

1% polyvinylpyrrolidone (PVP) (Fisher catalog no.: BP431-500)

β-mercaptoethanol (Fisher catalog no.: BP176-100)

CTAB buffer

RNase A

Proteinase K

Chloroform

Isoamyl alcohol

Phenol

7.5 M ammonium acetate

Isopropanol

70% ethanol

95% ethanol

**Procedure**

1. Freshly prepare **CTAB mixture** and use it on the same day:
2. Add **polyvinylpyrrolidone** as specified in the table to **CTAB buffer**. Warm it to 60°C. Just before adding the mixture to ground materials, add volume of **β-mercaptoethanol** as indicated by table to CTAB mixture.

| **2× CTAB (mL)** | **PVP (g)** | **β-mercaptoethanol (µL)** |
| --- | --- | --- |
| 0.5 | 0.02 | 2.5 |
| 5 | 0.2 | 25 |
| 20 | 0.8 | 100 |

1. Weigh 20 mg of silica-dried **plant tissues.** (Be careful not to cross-contaminate, e.g., Use new weighing paper each time and use tweezers dipped in 50% bleach solution to handle tissues.)
2. Grind tissue to a fine powder using mechanical grinding (e.g., using a bead beater with metal beads). Grinding can also be done with or without the aid of liquid nitrogen and/or autoclaved sand.
3. Wash the ground samples with freshly prepared **Sakaguchi isolation buffer**.

For 5 mL of CTAB mixture, add 10 µL of **RNase A** and 50 µL of **proteinase K** to the CTAB mixture (optional).

1. Add 500 µL of **CTAB mixture**. Grind samples for 2 s with the bead beater to mix well.
2. Incubate samples at 60°C for 30 min and transfer to 55°C for 1 h. Mix the contents by inversion occasionally.
3. Extract DNA using chloroform : isoamyl alcohol:
   1. For *Memecylon* and other Olisbeoideae taxa: Add 500 µL of **1 : 1 (24 : 1** **chloroform : isoamyl alcohol) : phenol** and mix well by inverting tubes.
   2. For *Tibouchina*: Add 500 µL of **24 : 1** **chloroform : isoamyl alcohol** and mix well by inverting tubes.
4. Centrifuge for 7 min at maximum speed (e.g., 13,200 rpm). Following centrifugation, there should be three layers (top: aqueous phase; middle: debris and proteins; bottom: chloroform). Go on to the next step quickly so the phases do not remix.
5. Estimate volume of the aqueous phase and transfer into a new labeled Eppendorf tube by pipetting off the aqueous phase, taking care not to suck up any of the middle or chloroform phases. Slow pipetting helps with this.
6. Discard the protein and chloroform layers in hazardous waste.
7. Add 0.08 volumes (relative to volume of aqueous phase) of cold 7.5 M **ammonium acetate**.
8. Add 0.54 volumes (using the combined volume of aqueous phase and added ammonium acetate) of cold **isopropanol (=2-propanol)**. Mix well.
9. Let sit in −20°C freezer for 30 min to overnight. Longer times will tend to yield more DNA, but also more contaminants. (If your DNA extraction is for Sanger sequencing, the recommendation is 30 min in freezer.)
10. Centrifuge for 5 min at maximum speed (13,200 rpm).
11. Pour or pipette off the liquid, being careful not to lose the pellet with your DNA.
12. Add 700 µL of cold **70%** **ethanol** and gently mix.
13. Centrifuge for 2 min at maximum speed (13,200 rpm).
14. Pour or pipette off the liquid, being careful not to lose the pellet with your DNA.
15. Add 700 µL of cold **95% ethanol** and gently mix.
16. Centrifuge for 1 min at maximum speed (13,200 rpm).
17. Pour or pipette off the liquid, being careful not to lose the pellet with your DNA.
18. Allow the pellet to dry completely:
    1. Air dry by inverting samples on a Kimwipe overnight OR
    2. Evaporate in the speed vac for 20 min
19. Resuspend samples with 50–100 µL of **nuclease-free water**. Allow to resuspend overnight in refrigerator before running a test gel of the DNA or a quantification assay.

**Stock Solutions:**

**CTAB:** for 1 L of CTAB buffer

100 mL of 1 M Tris, pH 8.0

280 mL of 5 M NaCl

40 mL of 0.5 M EDTA

20 g of CTAB (cetyltrimethyl ammonium bromide, Amresco catalog no.: 0833-1Kg)

**TE buffer:** for 1 L

[Final]

10 mM 10 mL of 1 M Tris, pH 8.0

1 mM 2 mL of 0.5 M EDTA

**1 M Tris, pH 8.0:** for 1 L

121.1 g Tris (Fisher catalog no.: BP152-5)

700 mL ddH_2_O

Dissolve Tris and bring to 900 mL

pH to 8.0 with concentrated HCl (will need ~50 mL)

Bring to 1 L

**0.5 M EDTA pH 8.0:** for 1 L

186.12 g of EDTA (Fisher catalog no.: BP120-1)

750 mL ddH_2_O

Add about 20 g of NaOH pellets

Slowly add more NaOH until pH is 8.0, EDTA will not dissolve until the pH is near 8.0

**5 M NaCl:** for 1 L

292.2 g of NaCl (Fisher catalog no.: BP358-10)

700 mL ddH_2_O

Dissolve and bring to 1 L

**Sakaguchi isolation buffer for washing ground leaf tissue prior to DNA extraction:**

This total volume can be used for 100 samples.

Materials:

PEG 10% 4.4 g

Sorbitol 0.35 M 2.8 g

Tris HCl, 1 M, pH 8, 50 mM, 3.5 mL

BSA 0.1% v/v 44 mg

β-mercaptoethanol 0.1% v/v 44 µL

Water 40 mL

Procedure:

Grind leaf tissue to powder; add isolation buffer (500 µL each time); incubate at 65°C for 1–2 h; spin for 4 min at 13,000 rpm; remove and discard supernatant. Repeat wash until clear solution. Then extract tissue pellet with standard CTAB extraction protocol.

**LITERATURE CITED**

Cullings, K.W. 1992. Design and testing of a plant-specific PCR primer for ecological and evolutionary studies. Molecular Ecology 1: 233–240.

Doyle, J. J., and J. L. Doyle. 1987. A rapid DNA isolation procedure for small quantities of fresh leaf tissue. *Phytochemical Bulletin* 19: 11-15.

Sakaguchi, S., D. Takahashi, H. Setoguchi, and Y. Isagi. 2018. Genetic structure of the clonal herb *Tanakaea radicans* (Saxifragaceae) at multiple spatial scales, revealed by nuclear and mitochondrial microsatellite markers. *Plant Species Biology* 33: 81–87.
