# Supplementary material for: A two‐tier bioinformatic pipeline to develop probes for target capture of nuclear loci with applications in Melastomataceae
Source: Appl Plant Sci. 2020 May 9;8(5):e11345. doi: 10.1002/aps3.11345 (PMC7249273; doi:10.1002/aps3.11345)
Supplement: Supplementary file 3 — APPENDIX S3. Percent on‐target reads, read counts, read depth, the number of loci for which sequences were recovered, and the number of potential paralogs for all species. [file APS3-8-e11345-s003.docx]

**APPENDIX S3.** Percent on-target reads, read counts, read depth, the number of loci for which sequences were recovered, and the number of potential paralogs for all species.

| Species | Mean percent on-target reads | Mean no. of reads mapped | Mean no. of total reads | Mean length (no zero, bp) | Maximum length (bp) | Mean (min–max) no. of templates with sequences | Mean no. of templates with sequences of 50% template length | Mean no. of potential paralogs |
| --- | --- | --- | --- | --- | --- | --- | --- | --- |
| *Memecylon amplexicaule* | 92.2 | 3,904,287 | 4,233,431 | 537 | 4098 | 315 | 201 | 8 |
| *Memecylon australissimum* | 84.7 | 3,419,850 | 4,060,390 | 551 | 4314 | 332 (329–334) | 218 (207–229) | 3 |
| *Memecylon bachmannii* | 86.6 | 3,717,926 | 4,306,749 | 539 | 4698 | 323 (290–347) | 209 (180–229) | 3 |
| *Memecylon caeruleum* | 83.0 | 2,431,869 | 2,928,378 | 562 | 4233 | 316 | 211 | 3 |
| *Memecylon calyptratum* | 88.9 | 3,422,508 | 3,848,062 | 548 | 4341 | 319 | 205 | 5 |
| *Memecylon capitellatum* | 91.3 | 3,739,783 | 4,096,460 | 484 | 3261 | 315 | 182 | 2 |
| *Memecylon clarkeanum* | 85.4 | 2,064,740 | 2,418,839 | 547 | 4341 | 314 | 208 | 2 |
| *Memecylon cuneatum* | 85.5 | 2,088,392 | 2,441,838 | 552 | 4233 | 310 | 206 | 2 |
| *Memecylon discolor* | 86.7 | 1,803,709 | 2,080,790 | 556 | 4341 | 298 | 199 | 3 |
| *Memecylon excelsum* | 91.4 | 3,319,047 | 3,630,570 | 493 | 4284 | 314 | 191 | 11 |
| *Memecylon flavescens* | 79.8 | 2,096,163 | 2,625,799 | 539 | 4230 | 303 | 198 | 8 |
| *Memecylon fruticosum* | 94.8 | 516,499 | 545,035 | 320 | 2055 | 229 | 85 | 0 |
| *Memecylon fuscescens* | 89.7 | 1,465,329 | 1,632,702 | 538 | 4236 | 286 | 189 | 0 |
| *Memecylon giganteum* | 88.8 | 950,246 | 1,070,182 | 536 | 4236 | 282 | 183 | 2 |
| *Memecylon gracillimum* | 84.5 | 2,295,063 | 2,715,446 | 556 | 4227 | 300 | 202 | 3 |
| *Memecylon grande* | 84.0 | 1,555,855 | 1,851,919 | 536 | 4338 | 311 | 200 | 6 |
| *Memecylon hookeri* | 83.9 | 1,066,598 | 1,271,186 | 553 | 4227 | 289 | 191 | 2 |
| *Memecylon kollimalayana* | 74.2 | 2,647,218 | 3,569,240 | 559 | 4230 | 317 | 212 | 7 |
| *Memecylon kosiense* | 78.5 | 871,178 | 1,109,818 | 564 | 4338 | 301 | 205 | 6 |
| *Memecylon lanceolatum* | 90.9 | 132,635 | 145,949 | 336 | 1134 | 111 | 39 | 0 |
| *Memecylon lawsonii* | 85.7 | 2,147,824 | 2,507,085 | 541 | 4236 | 306 | 198 | 3 |
| *Memecylon ligustrifolium* | 87.0 | 2,324,849 | 2,671,331 | 541 | 4338 | 314 | 200 | 2 |
| *Memecylon macrophyllum* | 80.8 | 12,442,53 | 1,540,585 | 552 | 4224 | 294 | 193 | 2 |
| *Memecylon malabaricum* | 84.8 | 3,648,524 | 4,304,074 | 517 | 3045 | 328 | 195 | 11 |
| *Memecylon maxwellii* | 92.9 | 1,995,292 | 2,146,671 | 495 | 4233 | 305 | 187 | 4 |
| *Memecylon natalense* | 82.9 | 1,794,677 | 2,127,129 | 556 | 4314 | 310 (289–334) | 207 (177–226) | 2 |
| *Memecylon obiculare* | 83.6 | 1,537,880 | 1,839,058 | 560 | 4227 | 302 | 204 | 6 |
| *Memecylon pauciflorum* | 95.3 | 2,637,300 | 2,767,224 | 477 | 4044 | 300 | 170 | 15 |
| *Memecylon petiolatum* | 87.4 | 3,167,293 | 3,624,123 | 546 | 4107 | 315 | 210 | 3 |
| *Memecylon procerum* | 84.1 | 1,367,321 | 1,626,165 | 545 | 4230 | 297 | 201 | 1 |
| *Memecylon randeriana* | 78.5 | 2,641,908 | 3,367,074 | 540 | 4230 | 326 | 212 | 4 |
| *Memecylon rhinophyllum* | 84.2 | 1,922,009 | 2,281,370 | 560 | 4230 | 307 | 205 | 5 |
| *Memecylon rivulare* | 89.6 | 2,329,518 | 2,600,174 | 531 | 4338 | 311 | 197 | 1 |
| *Memecylon rostratum* | 90.5 | 5,304,290 | 5,862,909 | 537 | 4404 | 331 | 211 | 12 |
| *Memecylon schraderbergense* | 92.0 | 1,453,811 | 1,580,013 | 513 | 4281 | 308 | 194 | 11 |
| *Memecylon scolopacinum* | 83.3 | 1,990,484 | 2,389,496 | 545 | 4233 | 306 | 202 | 8 |
| *Memecylon soutpansbergense* | 76.1 | 624,582 | 821,063 | 574 | 4314 | 290 | 203 | 3 |
| *Memecylon sylvaticum* | 83.6 | 1,226,418 | 1,466,754 | 543 | 4236 | 299 | 197 | 4 |
| *Memecylon symplociforme* | 42.0 | 80,427 | 191,535 | 370 | 1701 | 104 | 38 | 0 |
| *Memecylon terminale* | 82.3 | 3,430,933 | 4,168,096 | 546 | 4230 | 323 | 211 | 6 |
| *Memecylon umbellatum* | 78.4 | 994,675 | 1,268,057 | 546 | 4092 | 315 (301–328) | 206 (201–211) | 6 |
| *Memecylon urceolatum* | 85.6 | 2,081,844 | 2,432,431 | 550 | 4227 | 304 | 203 | 2 |
| *Memecylon varians* | 83.7 | 3,274,067 | 3,913,427 | 546 | 4227 | 321 | 217 | 6 |
| *Mouriri helleri* | 88.7 | 1,363,939 | 1,536,920 | 515 | 4215 | 310 | 187 | 3 |
| *Memecylon* sp1 | 77.6 | 1,387,452 | 1,788,073 | 554 | 4227 | 305 | 204 | 5 |
| *Memecylon* sp3 | 78.0 | 2,612,216 | 3,347,449 | 533 | 4221 | 329 | 209 | 8 |
| *Memecylon* sp5 | 81.7 | 2,647,977 | 3,241,139 | 544 | 4227 | 323 | 208 | 14 |
| *Memecylon* sp7 | 73.3 | 1,948,004 | 2,657,756 | 529 | 4104 | 317 | 202 | 14 |
| *Memecylon* sp8 | 75.1 | 1,807,753 | 2,406,206 | 574 | 4230 | 321 | 220 | 4 |
| *Memecylon* sp16 | 72.8 | 1,841,077 | 2,529,667 | 571 | 4230 | 319 | 220 | 5 |
| *Memecylon* sp18 | 86.0 | 2,298,362 | 2,671,128 | 554 | 4230 | 311 | 208 | 2 |
| *Memecylon wightii* | 87.4 | 9432,020 | 10,793,308 | 543 | 3876 | 322 (302–337) | 203 (170–221) | 14 |
| *Tibouchina* sp | 72.2 | 1,803,394 | 2,496,093 | 603 | 3837 | 318 | 267 | 25 |
| *Tibouchina aegopogon* | 81.4 | 1,452,294 | 1,774,516 | 550 | 4302 | 349 (295–366) | 261 (219–275) | 44 |
| *Tibouchina aegopogon* aff | 77.6 | 1,734,512 | 2,222,781 | 565 | 4062 | 351 (350–352) | 263 (261–265) | 64 |
| *Tibouchina albescens* | 78.8 | 1,953,563 | 2,459,476 | 567 | 5673 | 356 (351–363) | 271 (255–280) | 62 |
| *Tibouchina aegopogon* subsp. *angustifolia* | 84.6 | 2,287,098 | 2,703,812 | 540 | 3861 | 352 (306–381) | 256 (217–282) | 43 |
| *Tibouchina aspera* | 87.4 | 2,807,974 | 3,226,663 | 566 | 3969 | 348 (243–402) | 262 (154–323) | 14 |
| *Tibouchina barbigera* | 86.1 | 2,009,480 | 2,344,437 | 571 | 4512 | 364 (312–394) | 279 (222–316) | 51 |
| *Tibouchina barnebyana* | 82.4 | 2,082,837 | 2,527,492 | 576 | 3828 | 376 | 290 | 102 |
| *Tibouchina bipenicellata* | 88.0 | 2,087,095 | 2,374,970 | 554 | 3942 | 345 (295–381) | 256 (219–302) | 36 |
| *Tibouchina bruniana* | 90.1 | 2,477,729 | 2,751,906 | 507 | 3843 | 347 (332–355) | 248 (232–258) | 42 |
| *Tibouchina catharinae* | 89.8 | 1,143,231 | 1,273,246 | 527 | 3840 | 324 (312–335) | 235 (228–241) | 8 |
| *Desmoscelis villosa* | 71.2 | 1,388,336 | 1,949,948 | 656 | 3849 | 394 | 326 | 22 |
| *Tibouchina dissitiflora* | 91.1 | 761,891 | 835,058 | 541 | 3906 | 304 (287–321) | 232 (225–238) | 8 |
| *Tibouchina dubia* | 89.8 | 2,130,778 | 2,373,782 | 554 | 3846 | 351 | 265 | 97 |
| *Tibouchina duidae* | 90.9 | 980,512 | 1,074,689 | 522 | 3849 | 314 (299–328) | 230 (212–248) | 9 |
| *Tibouchina edmundoi* | 87.9 | 2,016,532 | 2,311,714 | 536 | 3951 | 356 (314–390) | 256 (225–278) | 33 |
| *Tibouchina exasperata* | 89.9 | 841,200 | 935,353 | 524 | 3528 | 331 | 242 | 31 |
| *Tibouchina fraterna* | 86.3 | 2,669,637 | 3,120,940 | 563 | 3963 | 365 (348–384) | 278 (263–287) | 37 |
| *Tibouchina gracilis* | 68.0 | 1,625,706 | 2,389,578 | 591 | 3831 | 320 | 262 | 21 |
| *Tibouchina johnwurdackiana* | 84.4 | 1,607,777 | 1,887,075 | 526 | 4083 | 297 (155–370) | 207 (68–289) | 35 |
| *Tibouchina karstenii* | 89.7 | 1,399,706 | 1,560,620 | 523 | 3831 | 346 (339–353) | 243 (239–247) | 28 |
| *Tibouchina kunhardtii* | 88.8 | 2,103,692 | 2,368,878 | 533 | 3825 | 329 | 240 | 11 |
| *Tibouchina llanorum* | 87.6 | 1,790,015 | 2,048,631 | 532 | 3840 | 328 (265–382) | 241 (197–280) | 35 |
| *Tibouchina mathaei* | 86.9 | 1,007,424 | 1,158,676 | 561 | 3834 | 355 | 259 | 25 |
| *Tibouchina melastomoides* | 82.7 | 2,257,343 | 2,699,790 | 549 | 4488 | 361 (340–372) | 267 (254–281) | 65 |
| *Tibouchina nigricans* | 82.1 | 2,726,292 | 3,311,535 | 568 | 4032 | 375 (355–402) | 285 (267–313) | 60 |
| *Tibouchina papyrus* | 83.2 | 2,581,112 | 3,072,283 | 549 | 3891 | 364 (338–397) | 271 (232–321) | 61 |
| *Tibouchina pogonanthera* | 90.1 | 480,002 | 532,843 | 528 | 3489 | 294 | 213 | 9 |
| *Tibouchina ramboi* | 89.9 | 2,291,996 | 2,549,243 | 618 | 3894 | 411 | 340 | 79 |
| *Tibouchina rosanae* | 91.3 | 446,670 | 489,211 | 513 | 4308 | 285 | 205 | 7 |
| *Tibouchina sipapoana* | 91.2 | 1,263,140 | 1,385,581 | 562 | 3897 | 332 | 258 | 11 |
| *Tibouchina* sp nov3 | 75.1 | 703,576 | 938,327 | 575 | 4494 | 335 (324–346) | 262 (257–267) | 46 |
| *Tibouchina* sp nov5 | 72.8 | 1,401,311 | 1,921,063 | 590 | 4347 | 360 (344–374) | 279 (262–292) | 58 |
| *Tibouchina* sp nov6 | 75.7 | 1,437,979 | 1,921,385 | 578 | 3903 | 362 (342–373) | 279 (260–289) | 52 |
| *Tibouchina* sp nov7 | 74.8 | 1,579,978 | 2,118,379 | 573 | 4719 | 353 (351–354) | 267 (263–271) | 71 |
| *Tibouchina* sp nov8 | 75.2 | 1,333,926 | 1,751,470 | 583 | 3834 | 349 (338–360) | 270 (266–274) | 61 |
| *Tibouchina* sp nov9 | 76.0 | 1,345,264 | 1,778,200 | 607 | 4386 | 366 (339–393) | 293 (265–321) | 54 |
| *Tibouchina spruceana* | 87.2 | 1,392,352 | 1,554,865 | 497 | 3615 | 294 (79–371) | 200 (10–262) | 27 |
| *Tibouchina striphnocalyx* | 84.7 | 4,424,764 | 5,222,221 | 574 | 3837 | 360 | 277 | 25 |
| *Tibouchina tuberosa* | 84.9 | 352,931 | 415,763 | 602 | 3075 | 335 | 264 | 35 |
| *Tibouchina verticillaris* | 83.1 | 2,167,190 | 2,604,409 | 533 | 4323 | 343 (248–371) | 249 (159–280) | 48 |
