# Supplementary figures and images for: A two‐tier bioinformatic pipeline to develop probes for target capture of nuclear loci with applications in Melastomataceae
Source: Appl Plant Sci. 2020 May 9;8(5):e11345. doi: 10.1002/aps3.11345 (PMC7249273; doi:10.1002/aps3.11345)

APPENDIX S5. Recovered sequence lengths for each category of template sequences.

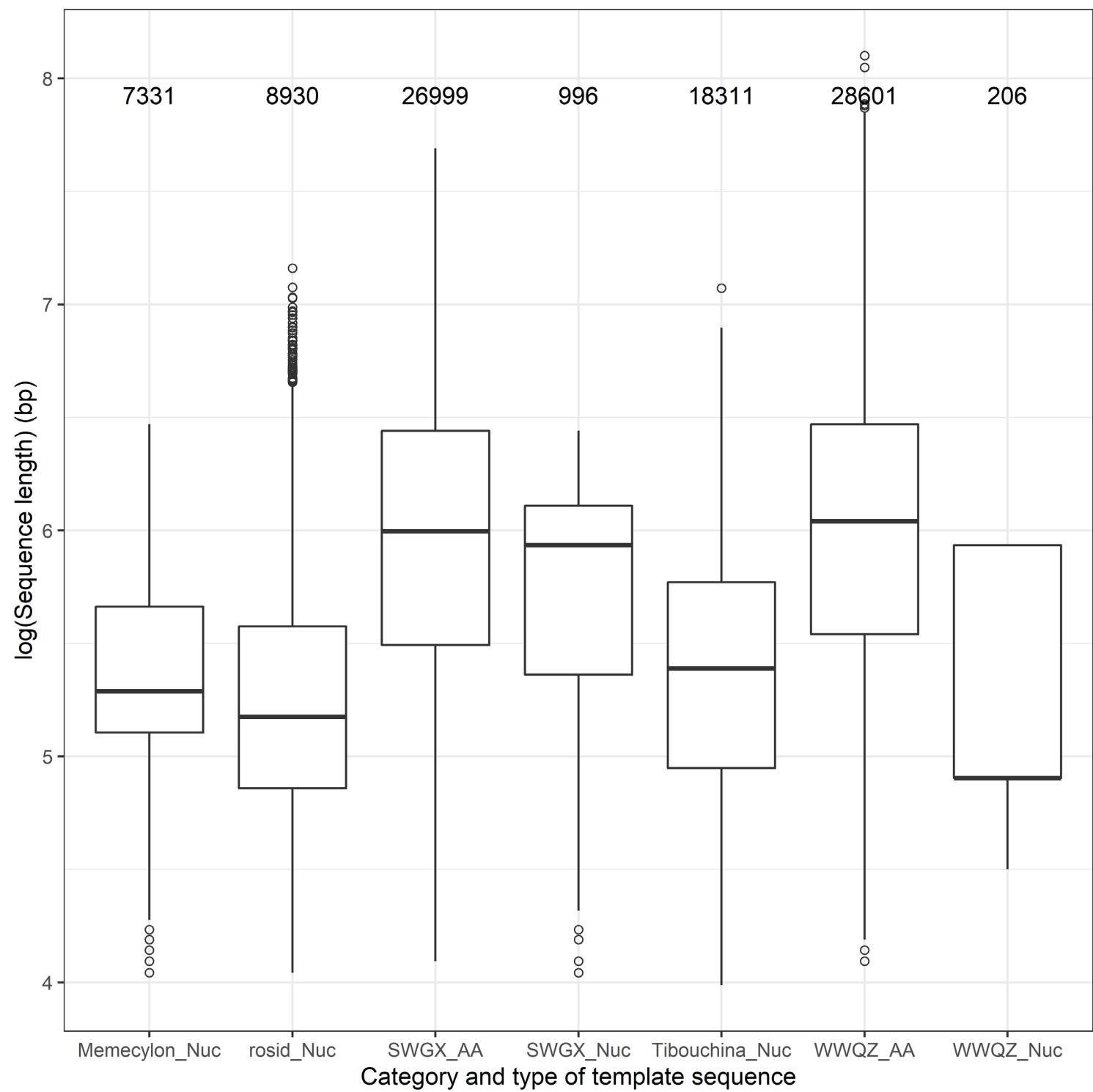

Supplement: Supplementary file 5 — APPENDIX S5. Recovered sequence lengths for each category of template sequences. [file APS3-8-e11345-s005.pdf]

**APPENDIX S6.** Read depth for template sequences.

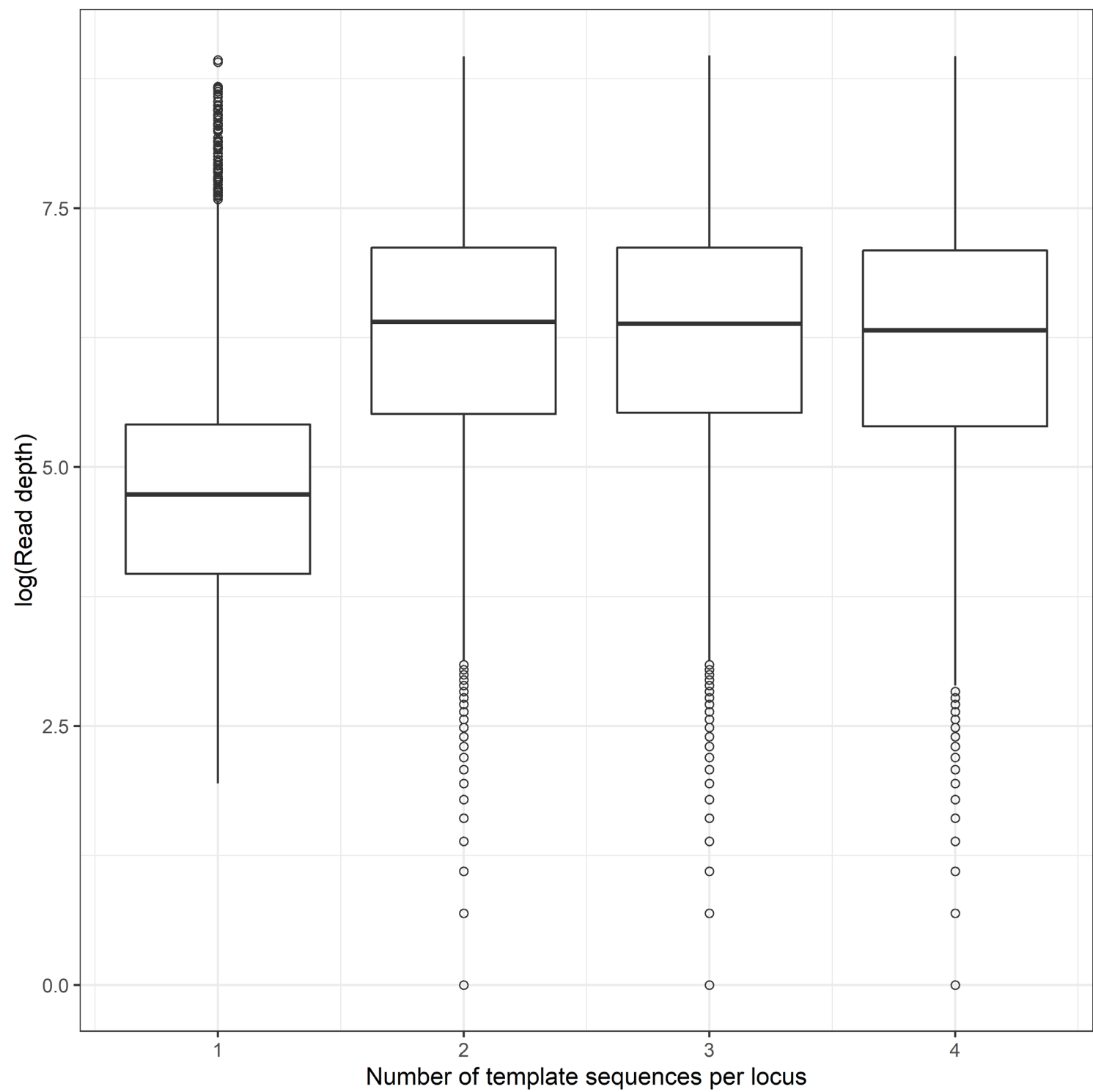

Supplement: Supplementary file 6 — APPENDIX S6. Read depth for template sequences. [file APS3-8-e11345-s006.pdf]
